# Comparative Assessment of Finite Element Modelling Strategies for Low-Velocity Impact of Thin GFRP Laminates

**DOI:** 10.3390/ma19214458

**Published:** 2026-10-23

**Authors:** Zhen Pei Chow

**Affiliations:** Department of Strength of Materials, Lodz University of Technology, 90-537 Lodz, Poland; zhenpei.chow@p.lodz.pl; Tel.: +48-790784159

**Keywords:** finite element modelling, modelling strategy, impact damage, composite laminate, low-velocity impact (LVI)

## Abstract

The primary objective of this study is to assess how different finite element modelling strategies influence the prediction of global impact response, internal damage evolution, and computational efficiency in thin glass fibre-reinforced polymer (GFRP) laminates subjected to low-velocity impact (LVI). A detailed reference model incorporating progressive intralaminar damage, cohesive-zone-based interlaminar delamination, and explicit representation of the experimental boundary condition was first established. Alternative boundary conditions, interface, and laminate representations were subsequently evaluated using nodal constraints, tiebreak contact formulations, thick-shell elements, and an equivalent single-layer formulation. Boundary idealisation was found to introduce localised peripheral delamination under low-energy impacts, while its influence on absorbed energy decreased with increasing impact energy, falling below 1.2% at 30 J. Replacing cohesive interfaces with tiebreak contact reduced computational cost by approximately 70% while preserving the overall impact response at lower impact energies, although the predicted damage morphology diverged when extensive delamination and perforation developed. Thick-shell and equivalent single-layer formulations further reduced model complexity but showed increasing limitations once progressive damage governed the response. The results demonstrate that similar global impact responses may be obtained from models with substantially different internal damage predictions, highlighting the need to select modelling strategies according to the response quantity of interest.

## 1. Introduction

Lightweight composite materials have become increasingly vital in engineering components due to their exceptional strength-to-weight ratios, design flexibility, and resistance to environmental degradation [1,2,3]. These properties have driven their widespread usage in automotive, aerospace, and marine structures, where minimising mass is crucial for enhancing performance and reducing energy consumption [4,5]. The multifunctionality and adaptability of these composites continue to stimulate research into structural performance, damage tolerance, and predictive modelling under increasingly demanding service conditions [6,7].

Despite their high strength-to-weight ratio and customizable mechanical performance, composite laminates—particularly thin fibre-reinforced polymer (FRP) structures remain susceptible to damage under out-of-plane loading [8]. Low-velocity impact events, often encountered during tool drops or accidental impacts during maintenance and handling, can introduce barely visible impact damage (BVID) and initiate matrix cracking, fibre fracture, and interlaminar delamination, particularly near stress concentrations and boundaries [9,10]. Thin laminated geometries are especially vulnerable, as their slenderness and bending-dominated response can lead to localised deformation and fracture failure even under relatively moderate impact energies [8,11,12]. Prior studies have addressed impact events at various energies from 5 to 50 J, across materials including fibre composites, hybrid laminates, and natural fibre composites [13,14,15]. However, the damage evolution remains complex, and accurate predictive modelling remains a critical challenge [9,16].

Previous experimental, numerical, and analytical studies explored the impact damage behaviour of GFRP laminates through theoretical, experimental, and numerical approaches, often aiming to characterise critical impact thresholds, delamination resistance, and energy absorption mechanisms [17,18]. Impact testing using instrumented drop-weight setups has been used to quantify force-time responses and damage size, while subsequent mechanical testing has been employed to evaluate residual strength across various layups and stacking sequences [19,20,21]. Numerically, Li et al. [22] explored the influence of damage initiation criteria (Hashin, Chang-Chang, Hou, Puck) and different damage evolution methods (linear and exponential strain/displacement) on low-velocity impacts. Similarly, Hu et al. [23] integrated the strain-based Hashin criterion, continuum damage mechanics, and cohesive zone modelling to predict the damage mechanisms of composite stiffened structures under low-velocity impact and compression after impact.

Most investigations remain limited to the non-perforation regime or sub-critical impact events, where only minor fibre fracture or matrix damage occurs, and complete layer separation is avoided [24,25]. Even recent finite element studies have primarily targeted low-to-moderate energy impacts, often omitting the more severe penetration and post-penetration behaviours that may arise in thin composite structures subjected to higher loads or repeated impacts [26]. Consequently, the predictive capability of many existing numerical models remains uncertain for thin composite structures experiencing severe impact events involving penetration and extensive delamination.

In the literature, some studies have meticulously incorporated detailed boundary fixtures such as rubber-tipped stoppers and clamped frames to replicate experimental drop-weight setups. Wang et al. [27] modelled glass-fibre metal laminates with multiple boundary constraint variations (from ASTM-standard clamp to round and square openings). The damage patterns were captured well in their FE simulation, owing to complex contact definitions and interface models. Similar accuracy was achieved by Pigazzini et al. [28] in their simulation of progressive damage in composite laminates using iso-geometric analysis. Falco et al. [29] employed a finite element modelling framework that explicitly incorporated rubber stoppers and a clamped steel frame in their simulation of low-velocity impact on GFRP laminates, achieving good agreement with experimental force-time curves and damage morphology. However, such detailed contact and fixture modelling resulted in significantly increased simulation times, especially when coupled with fine meshing, delamination, and contact definitions. Conversely, other studies simplify boundary condition representations using fixed edges or nodal pin constraints [30,31,32], which may be sufficient for capturing low-energy impacts but may not be suitable for modelling severe penetration or delamination propagation. These contrasting modelling strategies highlight the absence of a systematic assessment of how boundary representation influences both computational efficiency and impact damage prediction.

Besides boundary representation, the level of laminate discretisation and interface modelling also governs the predictive capability of impact simulations [33]. From a numerical modelling standpoint, simulating low-velocity impact on composites requires careful treatment of both material modelling and discretisation strategy. In thin-walled structures, the choice between solid elements, layered shells, or continuum shell formulations significantly affects the predicted bending stiffness, interlaminar stress fields, and damage propagation patterns [34]. Some models use layered shell elements to simulate large in-plane dimensions efficiently [35,36], allowing the overall impact response to be captured with reasonable accuracy, particularly at lower energy levels. Dogan et al. [37] investigated the effects of modelling delamination under impact with cohesive elements and tiebreak contact, and their results showed the importance of calibrating element size and contact parameters. The cohesive element approach, when combined with explicit deletion criteria, can be effectively used to simulate delamination but may require refined meshing and hourglass control [38]. Reduced-integration solid elements may additionally require hourglass control. The literature remains divided on best practices for simulating thin composite structures under impact, and the relative influence of individual modelling assumptions on different impact response quantities remains insufficiently clarified. It is hypothesised that not all aspects of a detailed impact model contribute equally to prediction accuracy. Specifically, boundary representation, interface formulation, and laminate discretisation are expected to influence different response quantities to varying degrees. It remains unclear whether artificial stress concentrations introduced by idealised supports alter only local damage patterns or also affect the global impact response. The novelty of this study lies in the systematic comparison of boundary condition, interface, and laminate representations within a consistent numerical framework. Their effects on global impact response, damage prediction, and computational efficiency were investigated. This approach helps identify modelling simplifications that remain suitable for low-velocity impact conditions of a thin GFRP plate.

This study aims to systematically investigate how boundary representation, interface formulation, and laminate discretisation influence the predicted response of thin GFRP laminates subjected to low-velocity impact. A detailed reference model comprising discrete solid plies, cohesive interlaminar layers, and explicitly represented boundary conditions is compared with a series of alternative modelling strategies, including simplified boundary representations (S1 and S2), tiebreak interfaces (S2-tb), thick-shell elements (S2-tb-TS), and an equivalent single-layer formulation (S2-ESL). By analysing impact responses at 10 J, 20 J, and 30 J, the study evaluates how these modelling choices affect global impact quantities, predicted damage morphology, and computational cost, while identifying the modelling features most relevant to specific impact responses.

The motivation for investigating these modelling strategies arises from the increasing computational demands associated with composite structures with more complex geometries, such as channel and cylindrical sections. Such models may require extensive mesh refinement, multiple contact definitions, and multi-component assemblies, resulting in substantially greater computational cost than conventional plate models. Similar challenges are evident in numerical studies of thin composite plates [39] and channel-section composite profiles [40], supported by corresponding experimental investigations [41,42]. The present study therefore provides a comparative assessment of commonly used modelling strategies and their associated trade-offs between computational efficiency, the prediction of global impact response, and numerical damage morphology. These findings are intended to provide practical guidance for selecting appropriate modelling strategies, while their applicability remains limited to the laminate configuration, material model, and impact conditions investigated herein.

## 2. Model Specifications

This section outlines the finite element (FE) model development and modelling pathways used to simulate low-velocity impact on thin composite laminates. All models were developed using the LS-DYNA explicit solver to capture the highly transient contact, damage initiation, and progressive failure associated with low-velocity impact.

**Figure 1:**
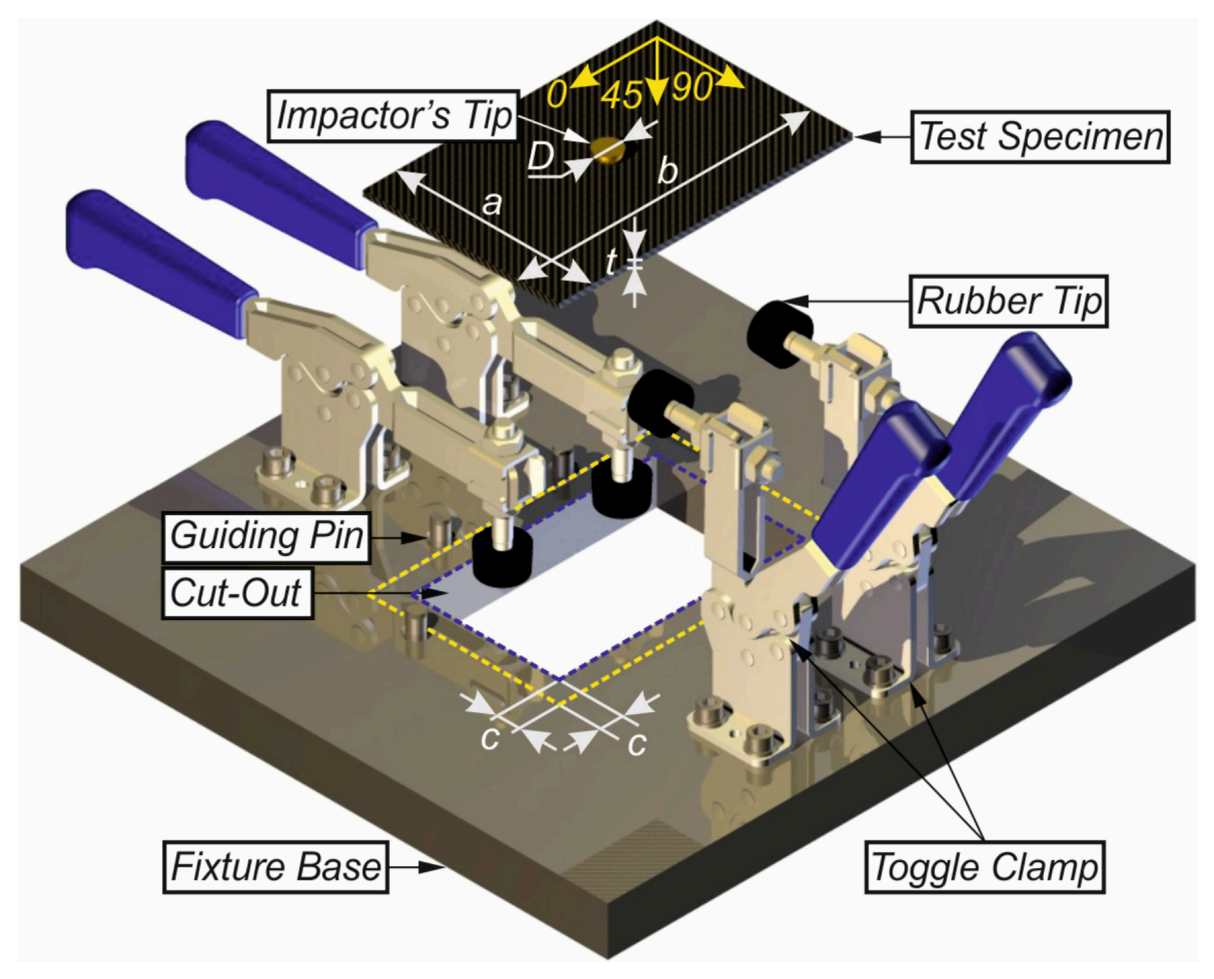
The experimental setup of the GFRP under LVI [43].

The FE model specifications are based on the existing experimental setup by Gliszczynski et al. [43] and on ASTM 7136 [44], as shown in Figure 1. The dimensions according to Figure 1 are: a = 150 mm, b = 100 mm, c = 12.5 mm, D = 12.7 mm, and t = 2 mm. Three impact energies were applied to a composite test specimen to achieve different stages of impact response: 10 J (indentation), 20 (damage), and 30 J (perforation). A validated detailed reference model serves as the baseline configuration, from which two types of model complexity are explored: boundary conditions and element type.

### 2.1. Detailed Reference Model

The detailed reference model (F), which is validated in a previous study [39], represents the most physically representative configuration and serves as the benchmark for evaluating subsequent alterations in the model. To summarise, the impacted plate consists of GFRP laminate in a symmetrically cross-plied quasi-isotropic [0/45/–45/90]s stacking sequence.

The GFRP laminate was modelled using solid elements with reduced integration (ELFORM 1) to sufficiently represent in-plane stiffness and capture intra-laminar failure behaviour. Each ply was modelled as an individual layer to resolve progressive intralaminar damage through LS-DYNA’s enhanced composite damage model MAT_054. The model incorporates the Chang–Chang failure criteria for tensile and compressive fibre failure and tensile and compressive matrix failure, expressed as follows:

(1) $$\begin{array}{c} {e_{f}}^{2}={\left(\frac{\sigma_{1}}{T_{1}}\right)}^{2}+\beta {\left(\frac{\tau_{12}}{S_{12}}\right)}^{2}-1\\ \sigma_{1}>0\\ {e_{c}}^{2}={\left(\frac{\sigma_{1}}{C_{1}}\right)}^{2}-1\\ \sigma_{1}<0\\ {e_{m}}^{2}={\left(\frac{\sigma_{2}}{T_{2}}\right)}^{2}+{\left(\frac{\tau_{12}}{S_{12}}\right)}^{2}-1\\ \sigma_{2}>0\\ {e_{d}}^{2}={\left(\frac{\sigma_{2}}{2S_{12}}\right)}^{2}+\left[{\left(\frac{C_{2}}{2S_{12}}\right)}^{2}-1\right]\frac{\sigma_{2}}{C_{2}}+{\left(\frac{\tau_{12}}{S_{12}}\right)}^{2}-1\\ \sigma_{2}<0 \end{array}$$

where *σ*_1_ and *σ*_2_ represent the normal stresses in the fibre and transverse directions, respectively, while *τ*_12_ denotes the in-plane shear stress. *T*, *C*, and *S* represent the corresponding tensile, compressive, and shear strengths, and *β* defines the shear contribution to fibre tensile failure. Damage initiates when the corresponding failure index reaches unity, followed by mode-dependent stiffness degradation, strength reduction, and eventual element deletion according to the prescribed failure parameters.

Interlaminar delamination was modelled using 8-noded, 4-point cohesive elements (ELFORM 19) to enable progressive initiation and propagation of delamination through a traction–separation law rather than instantaneous interface failure. These cohesive elements utilised the bilinear traction–separation law under MAT_138, with element deletion based on a mixed-mode failure formulation. The damage initiation separations and critical energy release rates under Mode I and Mode II are expressed as:

(2) $$\delta_{I}^{0}=\frac{\sigma_{max}}{E_{N}},\;\;\delta_{II}^{0}=\frac{\tau_{max}}{E_{T}}$$

(3) $$G_{IC}=\sigma_{max}\times \frac{\delta_{N}}{2},\;\;G_{IIC}=\tau_{max}\times \frac{\delta_{T}}{2}$$

where $E_{N}$ and $E_{T}$ represent the normal and tangential interface stiffnesses, respectively.

Hourglass deformation in the GFRP solids was controlled using the Flanagan–Belytschko stiffness formulation (IHQ=4) with a coefficient of 0.01. The formulation introduces artificial stiffness to suppress nonphysical zero-energy deformation modes associated with reduced-integration elements. Artificial hourglass energy was monitored throughout all simulations. Transient increases in hourglass energy were observed during the initial impact contact and local element erosion. For the detailed reference model, the hourglass energy remained stable throughout the simulation without sustained growth during the impact response, indicating acceptable numerical stability. Moreover, the predicted global impact response showed good agreement with the experimental results.

The material properties of the unidirectional GFRP lamina are shown in Table 1. Lamina elastic properties (*ρ*, *E*_1_, *E*_2_, *G*_12_, *G*_23_, *G*_31_, *ν*_12_) and strength properties (*T*_1_, *C*_1_, *T*_2_, *C*_2_, *S*_12_) are based on the original experimental study by Gliszczynski et al. [43]. Additional parameters that dictate ply softening (*ALPH*, *BETA*, *SOFT*, *FBRT*, *YCFAC*) and failure criteria (*DFAILT*, *DFAILC*, *DFAILM*, *DFAILS*, *EFS*) were adopted from the literature or numerically adjusted during the previous model development [39].

The cohesive zone properties were adopted from previous experimental efforts and shown in Table 2 below. Mode-I delamination properties (*G_IC_*, *σ_max_*, *E_N_*) are based on findings in [45] and [46], while Mode-II delamination properties (*G_IIC_*, *τ_max_*, *E_T_*) are adopted from [47]. The normal and tangential interface stiffnesses ($E_{N}$, $E_{T}$) were determined using the penalty stiffness approach proposed by Turon et al. [48], expressed as:

(4) $$K=\frac{\alpha E_{3}}{t}$$

where α=50 is the penalty factor, $E_{3}=8.1$ GPa is the assumed through-thickness Young’s modulus, and *t* = 0.25 mm is the individual ply thickness. The same stiffness was assigned in the normal and tangential directions.

The equations for MAT_054 and MAT_138 are consistent with their formulations in the LS-DYNA material manual; the Flanagan–Belytschko type corresponds to IHQ = 4. Turon’s stiffness relationship is also confirmed by the original publication [48].

Elements were discretised based on the plate geometry and the boundary conditions. Each ply and cohesive interface was modelled with a single element through thickness, resulting in a total model thickness of 2.07 mm, accounting for the cohesive layers of 0.01 mm each. Overall mesh sensitivity assessment was carried out, and an element size of 1.0 mm was chosen. Further details can be found in Section 2.4 about numerical verification.

**Figure 2:**
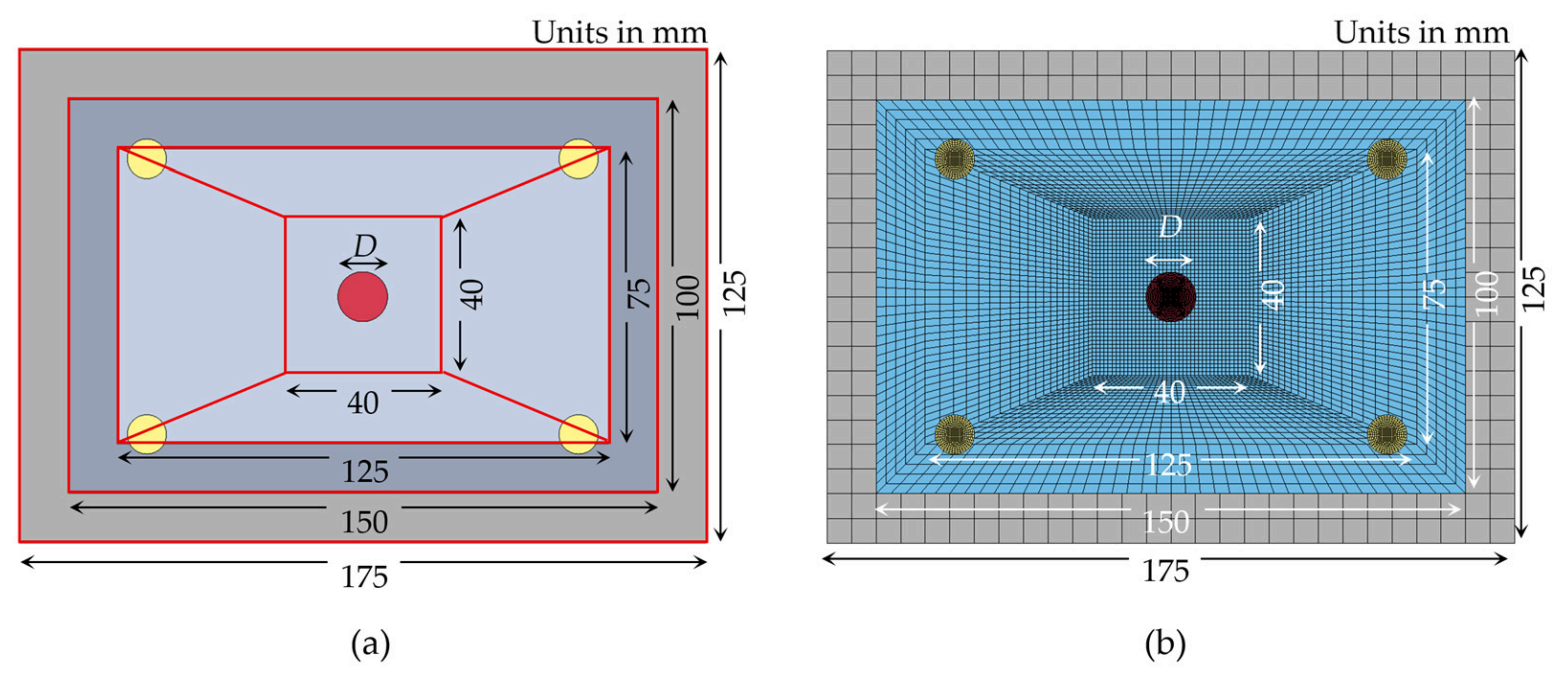
The meshing strategy of the F model from the top view: (**a**) mesh discretisation, (**b**) meshed model.

The detailed meshing strategy is shown in Figure 2. The GFRP panel measuring 150 mm × 100 mm lies on top of a fixed 175 mm × 125 mm rectangular frame with a 125 mm × 75 mm rectangular cutout. These rectangular frame dimensions act as the respective bounds for element size transition. Finer elements were used in high-damage zones, while coarser meshes were implemented further from the impact zone to reduce computational time. A minimum element size of 1 mm was applied to a central 40 mm × 40 mm region near the expected impact area to sufficiently resolve damage initiation and propagation. The elements furthest away and on the rectangular frame were meshed with an average size of 3 mm. An area of intermediate element size was implemented within the bounds of the rectangular frame opening of 125 mm × 75 mm, ensuring that a gradual transition is achieved between the central and furthest regions.

**Figure 3:**
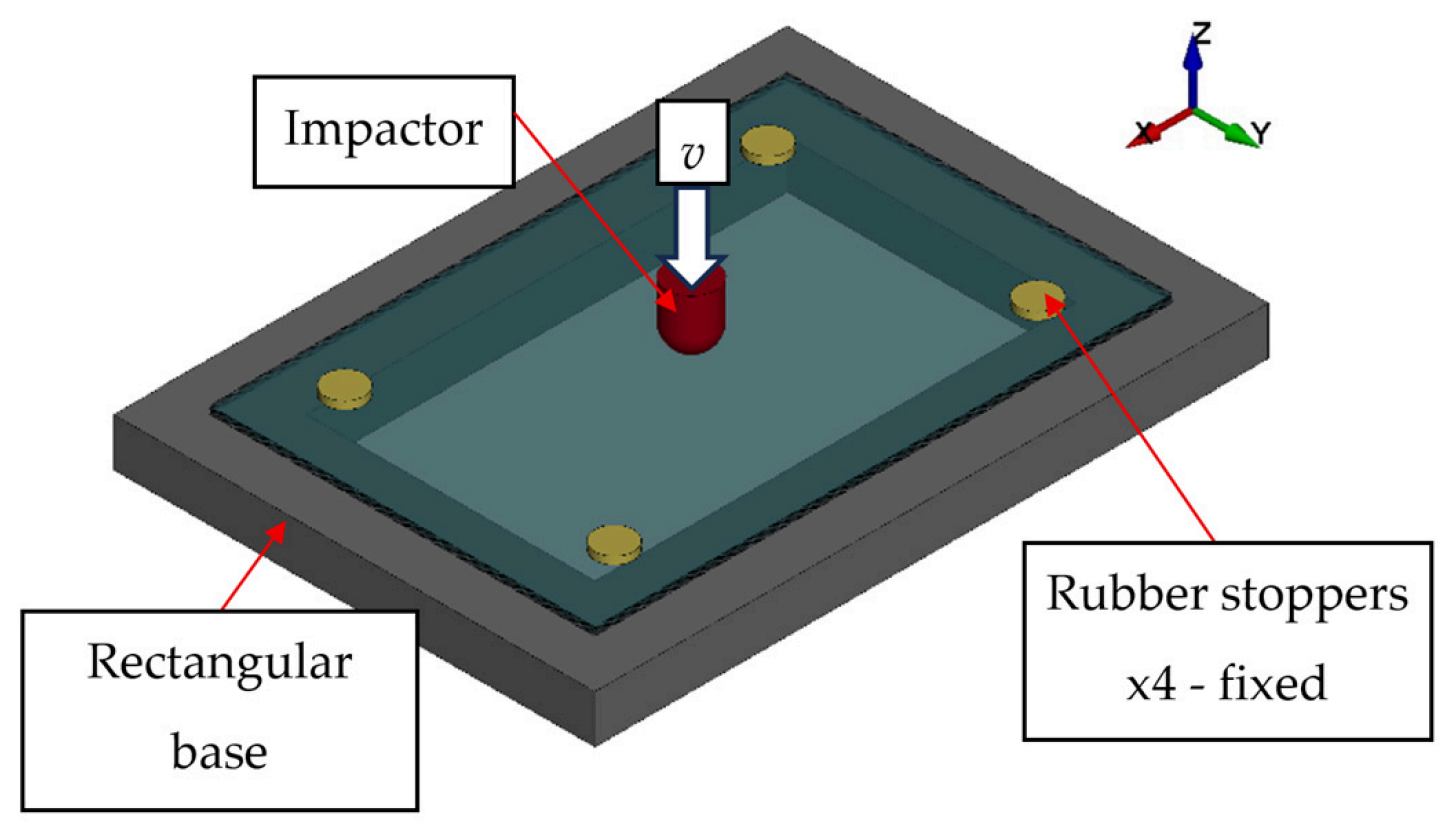
The boundary conditions for the F model.

Boundary conditions (Figure 3) included a detailed experimental replication using a rigid steel rectangular base with a rectangular cutout, four fixed rubber stoppers, and a rigid hemispherical impactor. These were defined using MAT_020 in LS-DYNA. The GFRP panel was modelled exactly on top of the fixed steel support, with their respective nodes overlapping, but not merged. The rubber stoppers, modelled as rigid cylinders with a diameter of 10 mm, were fixed in place to provide lateral support while allowing some deformation near the edges. The hemispherical impactor had a diameter of 12.7 mm and a mass of 3.05 kg and was initialised at velocities corresponding to nominal impact energies of 10 J, 20 J, and 30 J (approximately 2.57, 3.64, and 4.46 m/s, respectively).

Two AUTOMATIC_SURFACE_TO_SURFACE contacts were defined—between the composite plate and both the steel base and rubber stoppers. An ERODING_SURFACE_TO_SURFACE contact was defined between the impactor and the top surface of the plate to account for localised damage and material erosion. The same eroding contact strategy was also applied between each GFRP ply to simulate interlaminar failure realistically. This F model configuration establishes the baseline for subsequent simplification in boundary conditions and element formulations analysed in subsequent sections.

The representative models were deliberately constructed so that each successive model modifies only a single aspect of the preceding configuration. This sequential abstraction enables the individual influence of boundary representation, interface formulation, and laminate discretisation on impact response to be isolated and quantified without introducing multiple simultaneous modelling changes.

### 2.2. Numerical Verification

A preliminary mesh sensitivity assessment was performed using in-plane element sizes of 0.5, 1.0, 2.0 and 5.0 mm. The coarser meshes exhibited severe element distortion and numerical instability, whereas further refinement below 1.0 mm resulted in increasingly localised damage and reduced peak impact forces. Conventional mesh convergence could therefore not be demonstrated within the investigated range. Consequently, the 1.0 mm mesh was retained as a practical compromise between numerical stability, computational efficiency and agreement with the available experimental response. The remaining mesh sensitivity, particularly regarding progressive damage and failure predictions, is acknowledged as a limitation of the numerical model.

The hourglass energy was evaluated to assess the numerical behaviour of different models. Table 3 presents the hourglass-to-internal energy (HG/IE) ratios at the time of maximum hourglass energy for each model under each impact energy. The evaluated ratios range from 0 to 7%, excluding the S2-tb-TS model at 30 J. Although higher instantaneous HG/IE ratios (approximately 20 to 30%) were observed during the initial loading stage, these brief increases were attributed to relatively low internal energy at the beginning of impact, which amplifies the calculated ratio. Regarding the S2-tb-TS model at 30 J, severe element distortion occurred during the unloading stage after the peak impact force, which resulted in negative internal energy. Consequently, the corresponding HG/IE ratio was omitted as it was not meaningful. The S2-ESL model had zero hourglass energy because fully integrated shell elements were used.

### 2.3. Boundary Condition Representation

This section explores the influence of boundary condition (BC) representation on the impact behaviour of thin composite plates. Three finite element models are compared: the F model, as mentioned in Section 2.1, replicates the experimental setup with high agreement, with a supporting bottom rectangular plate and four rubber stoppers.

**Figure 4:**
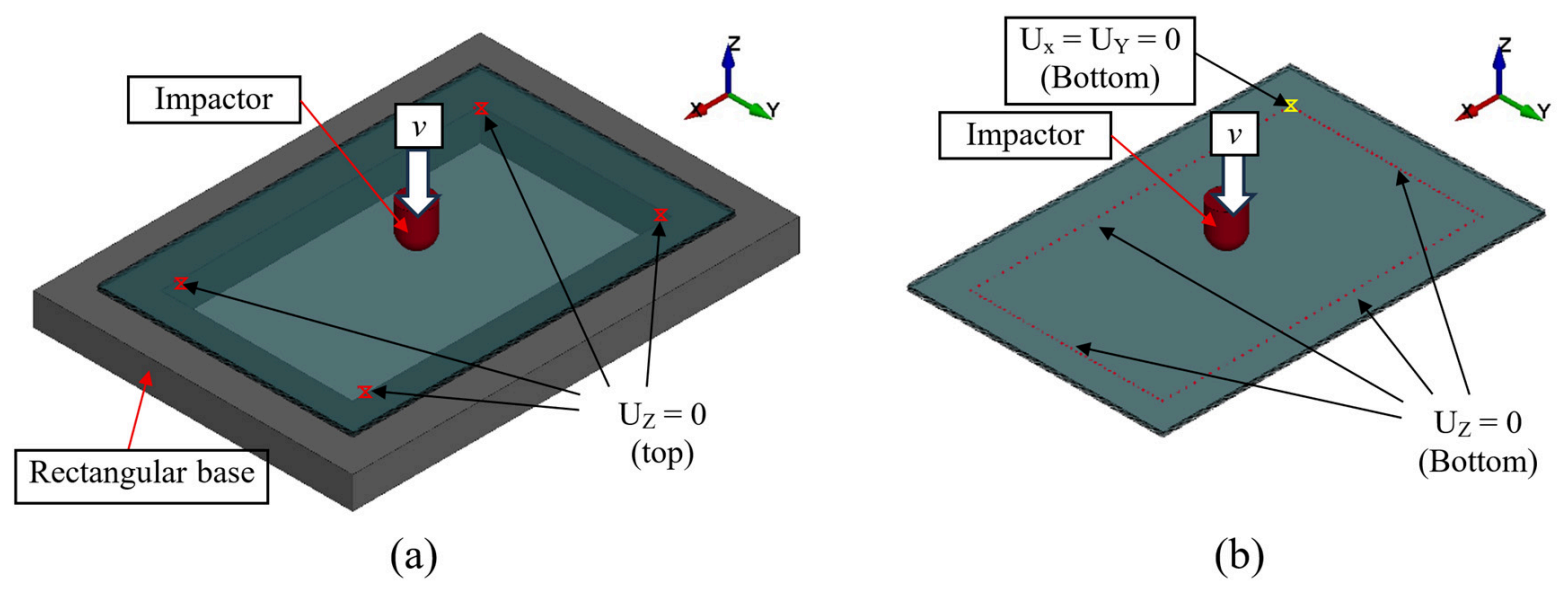
The boundary conditions for (**a**) the S1 model and (**b**) the S2 model.

Based on Figure 4, the S1 model represents the four rubber stoppers as 4 individual vertical nodal constraints (U_Z_ = 0) at equivalent locations where the centre of the four rubber stoppers lay. The 4 nodes constrained were the top nodes of the topmost layer of the GFRP. The S2 model provides an alternative boundary representation by eliminating the bottom plate entirely and applying distributed vertical nodal constraints (U_Z_ = 0) along the inner projected boundary lines where the plate would nominally contact the support. The distributed nodal constraints were applied to the bottom nodes of the bottommost layer of the GFRP. An additional constraint was applied on one of the nodes at the corner of this boundary line at the bottom of the bottommost node to prevent sideways motion (U_x_ = U_Y_ = 0). These boundary representations were introduced to isolate the influence of support idealisation on global impact response and localised damage evolution while progressively decreasing computational complexity [27,38]. The present work systematically quantifies the predictive capabilities of these assumptions over a wider range of impact severities, including perforation.

### 2.4. Laminate Representation

This section investigates how different laminate representations influence the predictive capability of impact simulations. Two derivative models were developed. The S2-tb model, in which cohesive elements are removed, uses tiebreak contacts (AUTOMATIC_ONE_WAY_SURFACE_TO_SURFACE_TIEBREAK) between adjacent plies. The tiebreak model incorporates the Dycoss Discrete Crack model with reduced complexity and computational cost compared to explicit cohesive elements. The traction and failure parameters were calibrated to remain consistent with the cohesive-zone formulation, enabling a direct assessment of the influence of interface representation independent of material calibration. With the removed cohesive elements, the GFRP layers are now directly stacked on top of each other, with their nodes at the same location, but not merged. Without the individual cohesive layer thickness of 0.01 mm, the total model thickness of the S2-tb model is 2 mm.

The second iteration of laminate representation is the S2-tb-TS model, which incorporates previous tiebreak interfaces while further replacing solid GFRP plies with thick shell elements with assumed strain reduced integration (ELFORM 5). Each thick shell layer represents a full ply with integrated composite properties using MAT_054 and layer-wise integration. The rest of the parameters and boundary conditions were kept the same as the S2 model. This abstraction evaluates whether through-thickness discretisation remains necessary to obtain good agreement in predicting impact-induced damage.

Finally, S2-ESL further represents the model by integrating the eight plies as a single shell layer. The single laminate represented layer keyword uses through-thickness integration to define the thickness and angle orientation for each individual ply. Because the laminate is represented as a homogenised shell, explicit interlaminar interfaces are omitted, thereby eliminating the possibility of progressive delamination. All the models implemented are summarised below in Table 4.

## 3. Results and Discussion—BC Representation

The reference F model was assessed against the experimental results at all three impact energies. Peak force and maximum displacement showed maximum deviations of 10.1% and 7.2%, respectively. Larger relative differences were obtained for absorbed energy at the lower impact energies. At 10 J, for example, the relative difference in the final absorbed energy reaches 74.1%; however, this corresponds to an absolute difference of 1.64 J, or 16.4% of the incident impact energy. As shown by the complete force- and energy-time histories in Section 3.1, the discrepancy develops mainly during unloading and rebound, while the loading-stage response remains closely reproduced. The complete response histories therefore provide important context when interpreting the terminal absorbed-energy value alone. Direct percentage comparison of damage area was not adopted because the numerical and experimental damage measures are based on different definitions and are not strictly equivalent observables.

### 3.1. Influence of Boundary Representation on Impact Response

The influence of boundary representation was first assessed by comparing the time histories of contact force, impactor displacement, absorbed energy, and impactor velocity against experimental measurements. The absorbed energy was determined from the impactor kinetic energy obtained from the MATSUM output in LS-DYNA, expressed as:

(5) $$E_{abs}=E_{KE,initial}-E_{KE,final}$$

where $E_{KE,initial}$ and $E_{KE,final}$ represent the initial and residual kinetic energies of the impactor, respectively. The resulting energy loss provides an estimate of the energy transferred to the impacted structure, with the final value evaluated after impactor separation.

These responses represent the principal global indicators governing impact performance and provide an initial assessment of whether detailed boundary modelling is required for sufficient prediction.

**Figure 5:**
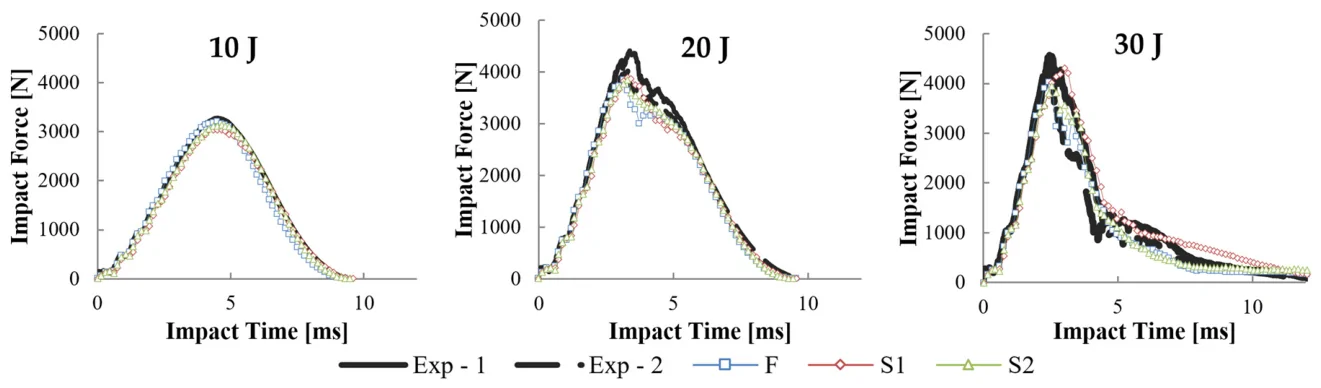
Force-time curves for experiments and FE models with different boundary representations. (colour should be used).

**Figure 6:**
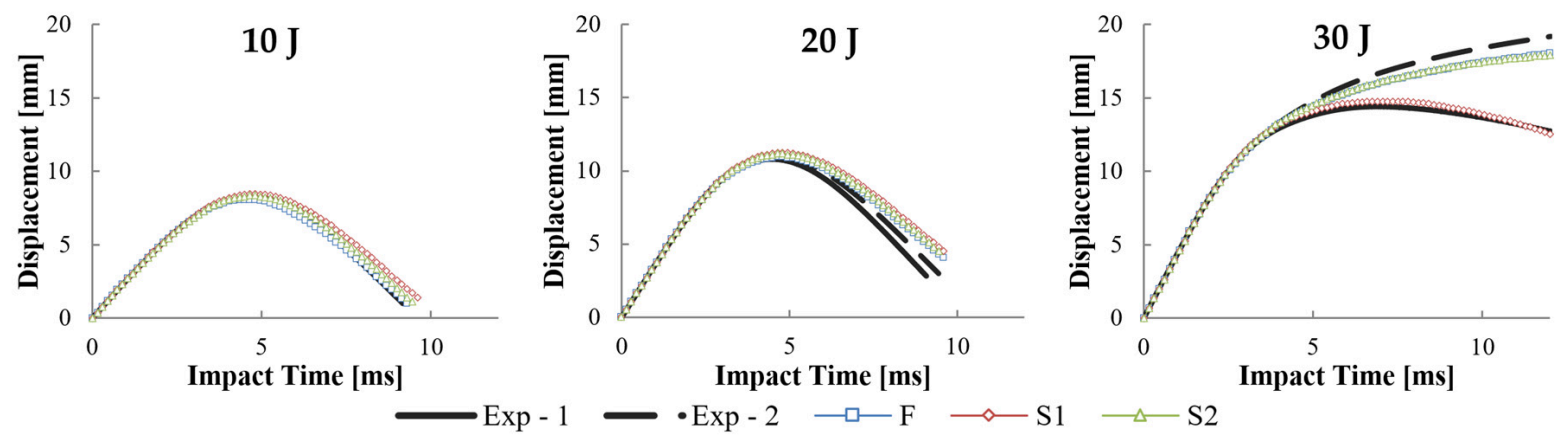
Displacement-time curves for experiments and FE models with different boundary representations. (colour should be used).

**Figure 7:**
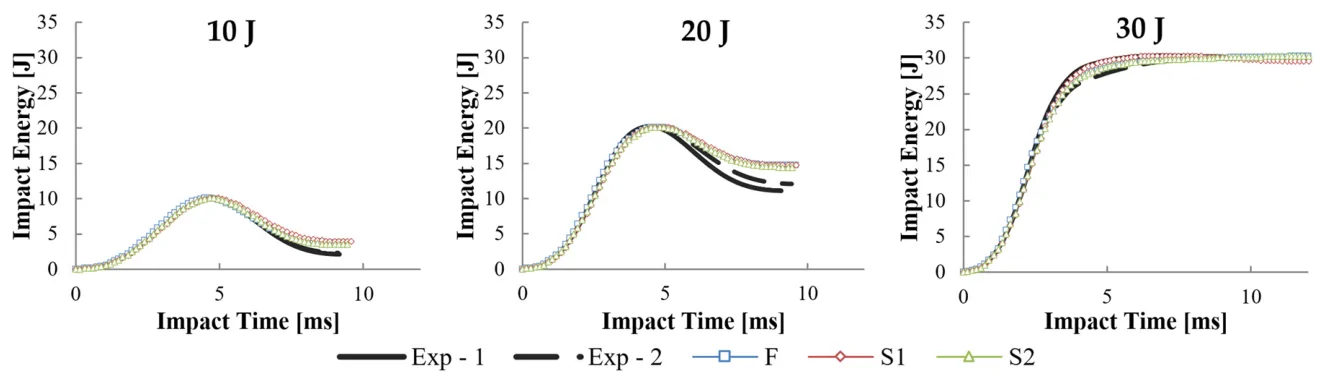
Energy-time curves for experiments and FE models with different boundary representations. (colour should be used).

**Figure 8:**
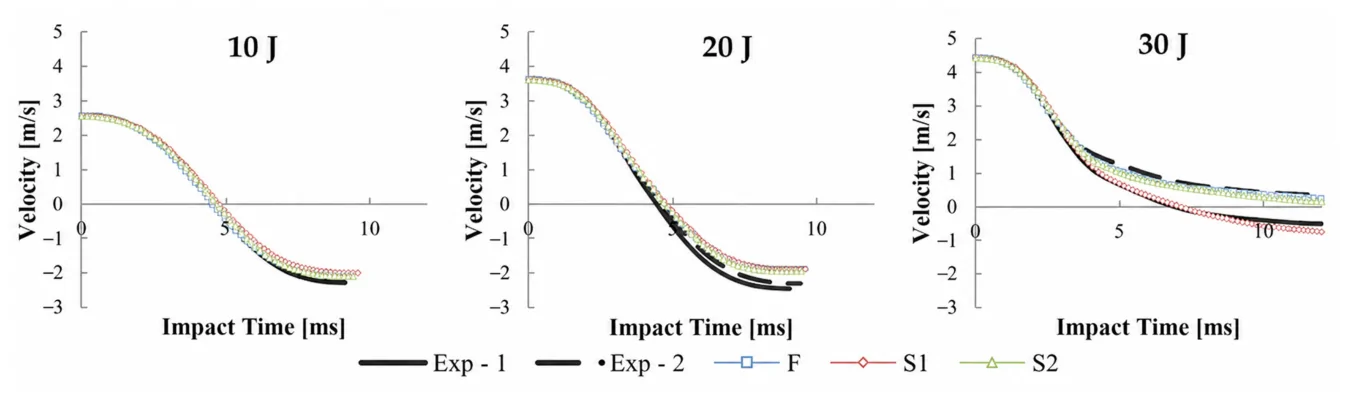
Velocity-time curve for experiments and FE models with different boundary representations. (colour should be used).

Having established the performance and limitations of the reference model, the influence of alternative boundary representations was assessed through comparison with S1 and S2 (Figure 5, Figure 6, Figure 7 and Figure 8). The almost identical histories at 10 J indicate that localised modifications of the boundary representation do not significantly influence the global structural response under low-energy impact. The global response remains largely insensitive to boundary representation at 30 J, suggesting that the dominant load path is governed by the localised impact region rather than by the supporting fixture.

**Figure 9:**
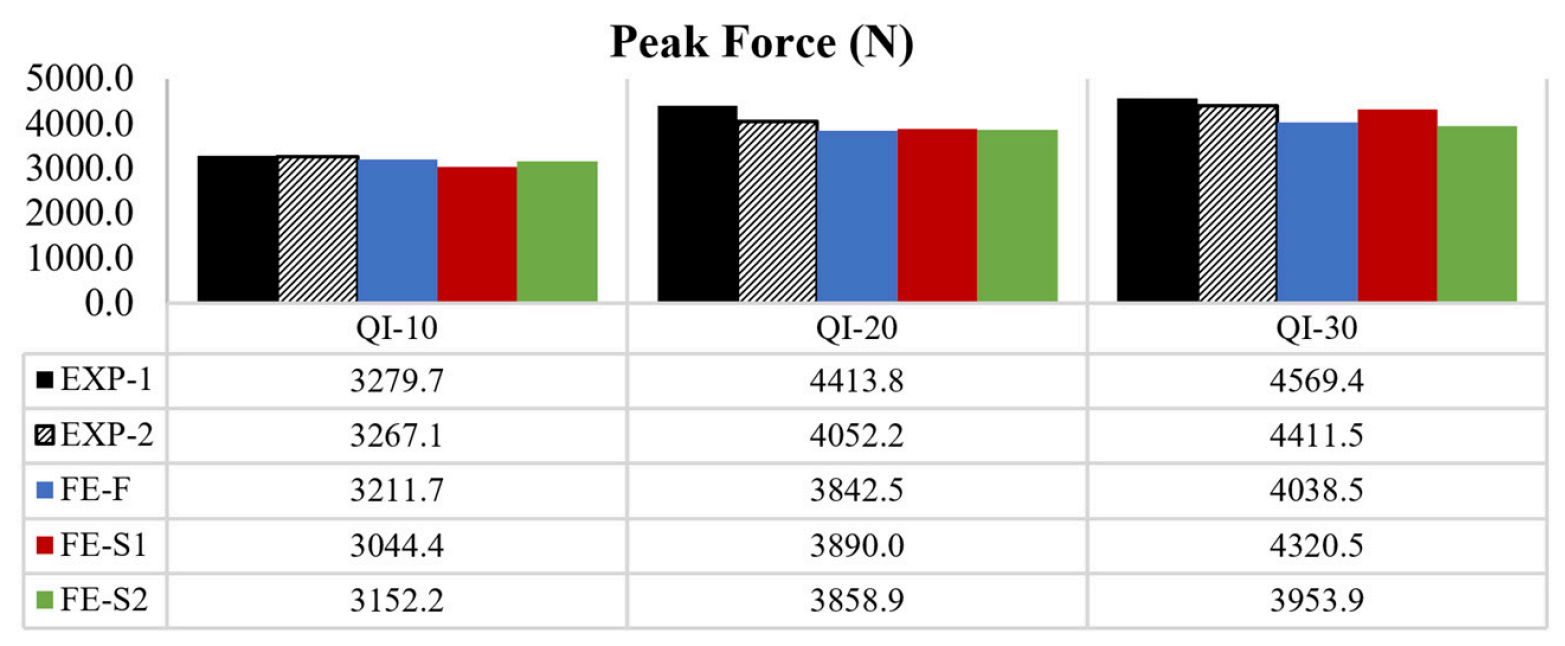
Peak Force for experiments and FE models with different boundary representations.

**Figure 10:**
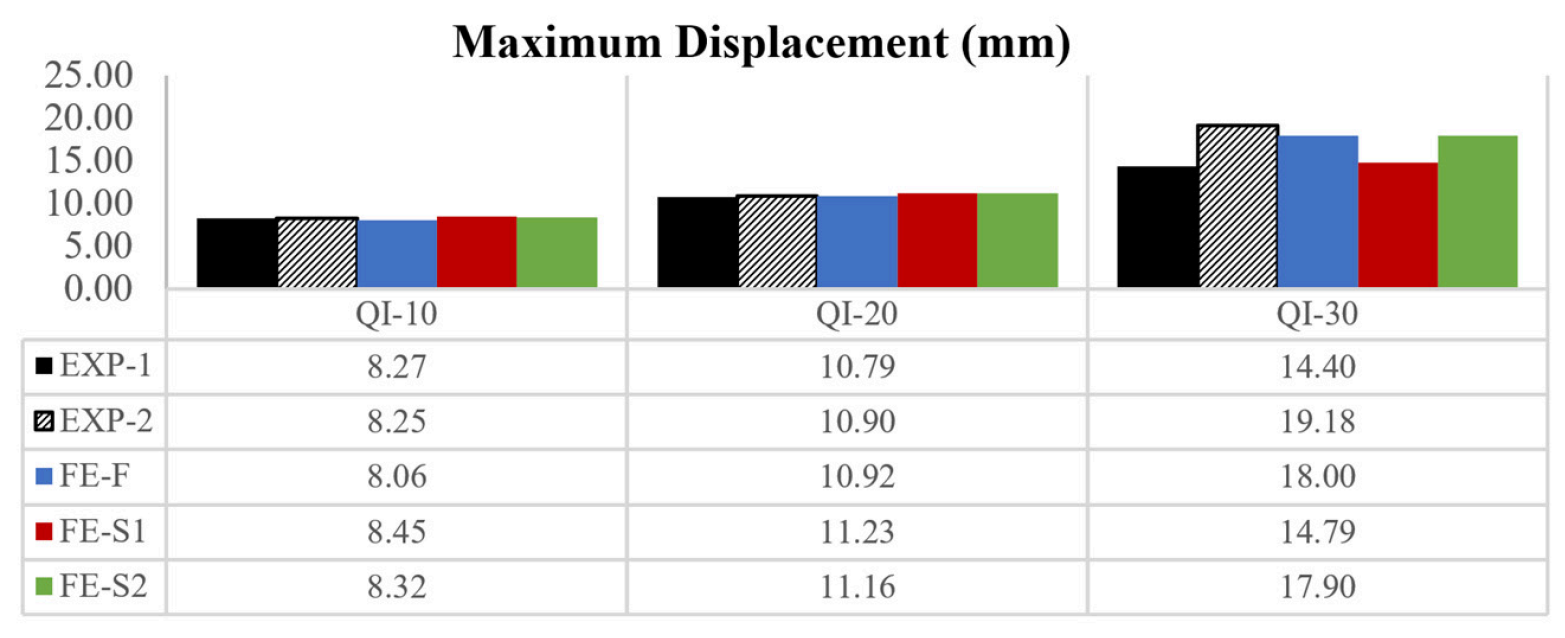
Maximum impactor displacement for experiments and FE models with different boundary representations.

**Figure 11:**
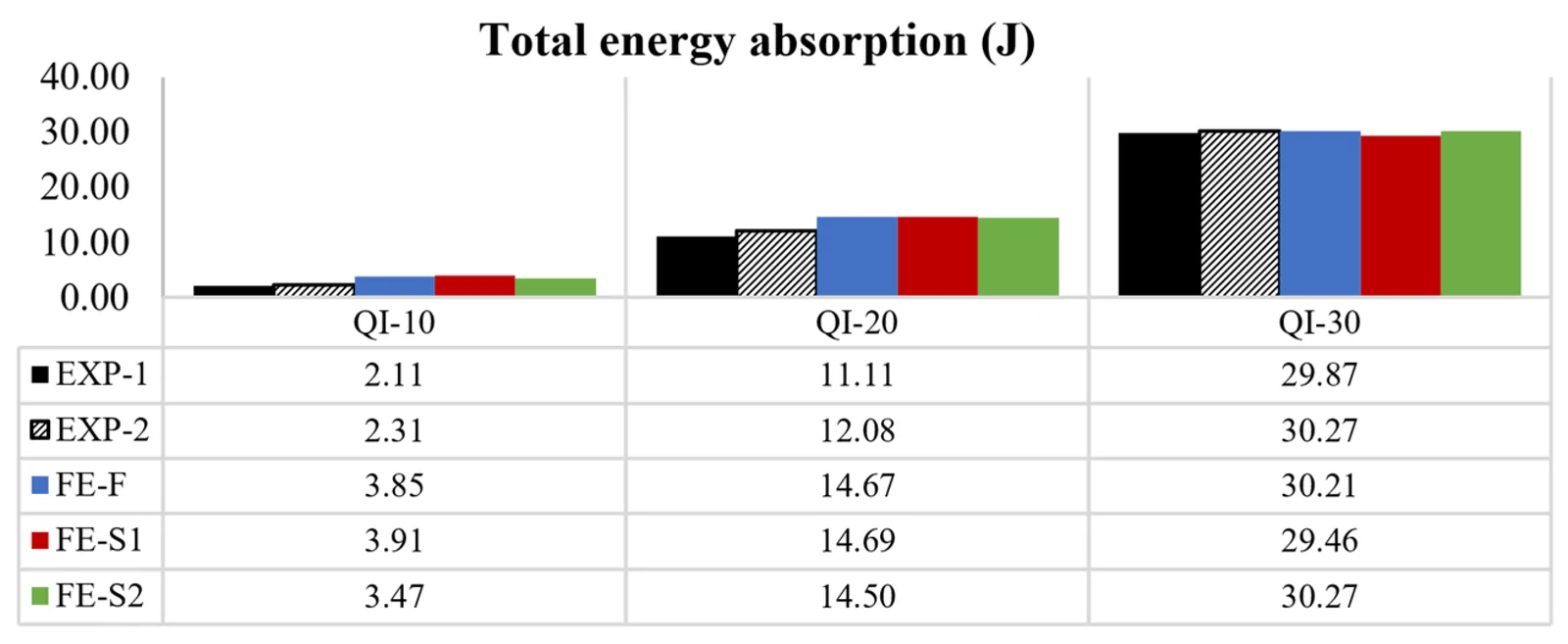
Total energy absorption for experiments and FE models with different boundary representations.

**Figure 12:**
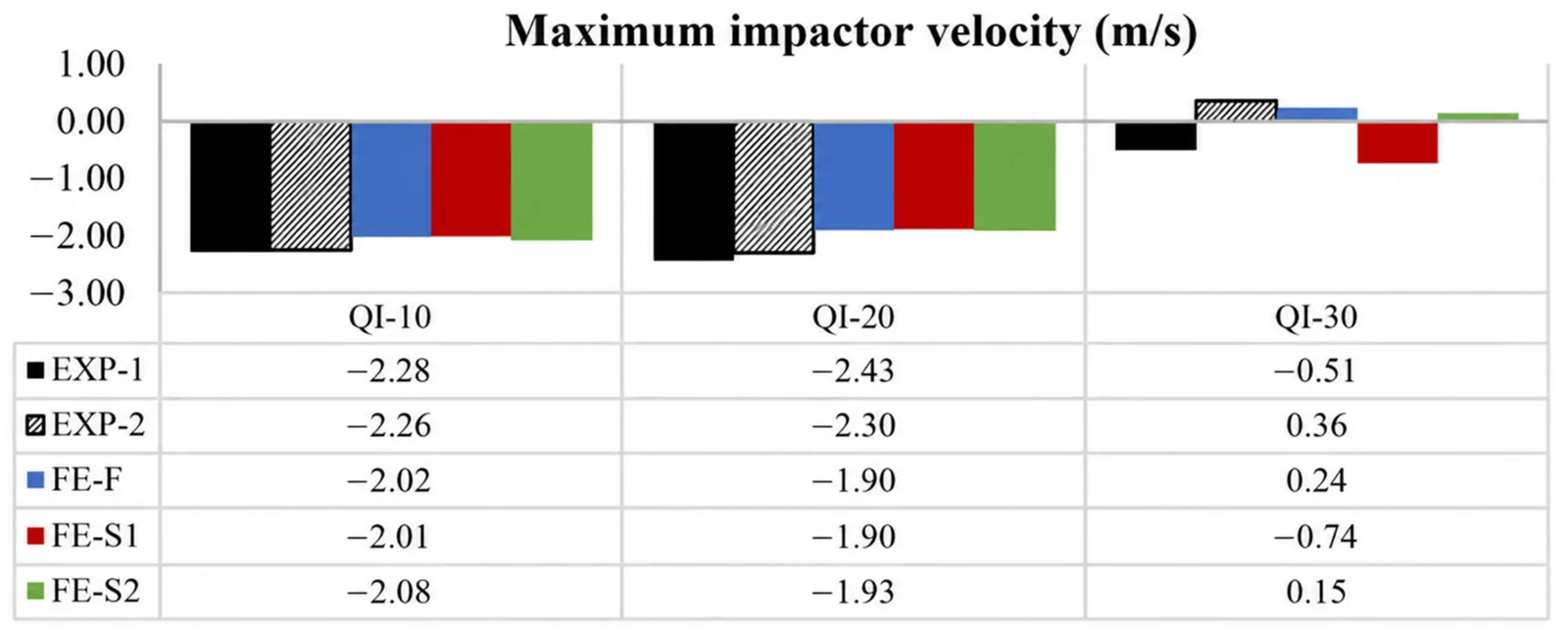
Maximum impactor velocity for experiments and FE models with different boundary representations.

According to Figure 9, Figure 10, Figure 11 and Figure 12, peak force remained within approximately 12% of the experimental mean despite progressive abstraction of the boundary representation, confirming that load transfer is largely unaffected by the modelling assumptions. The variation in maximum impactor displacement of each model lies well between the Exp-1 and Exp-2 range, despite the unpredictable impact damage. The close agreement in absorbed energy and maximum impactor velocity demonstrates that global energy dissipation is primarily governed by localised damage beneath the impactor, signifying the agreement level of each model.

### 3.2. Effects of BC Representation on Damage Morphology

**Figure 13:**
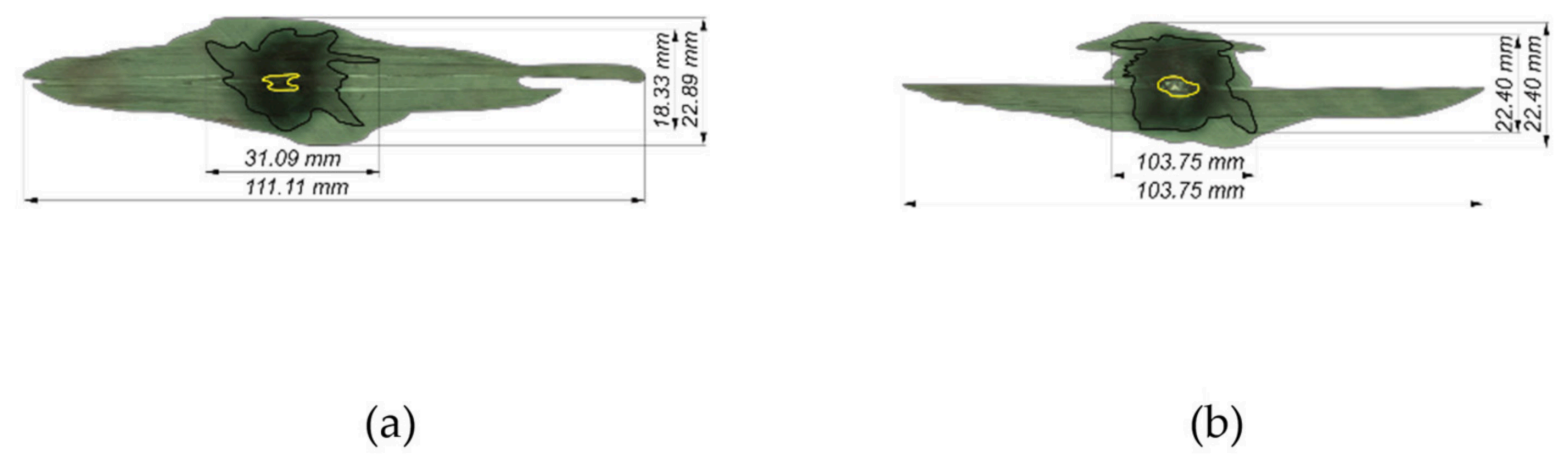
Experimental damage morphology of GFRP laminates subjected to 30 J impact: (**a**) EXP-1, (**b**) EXP-2.

**Figure 14:**
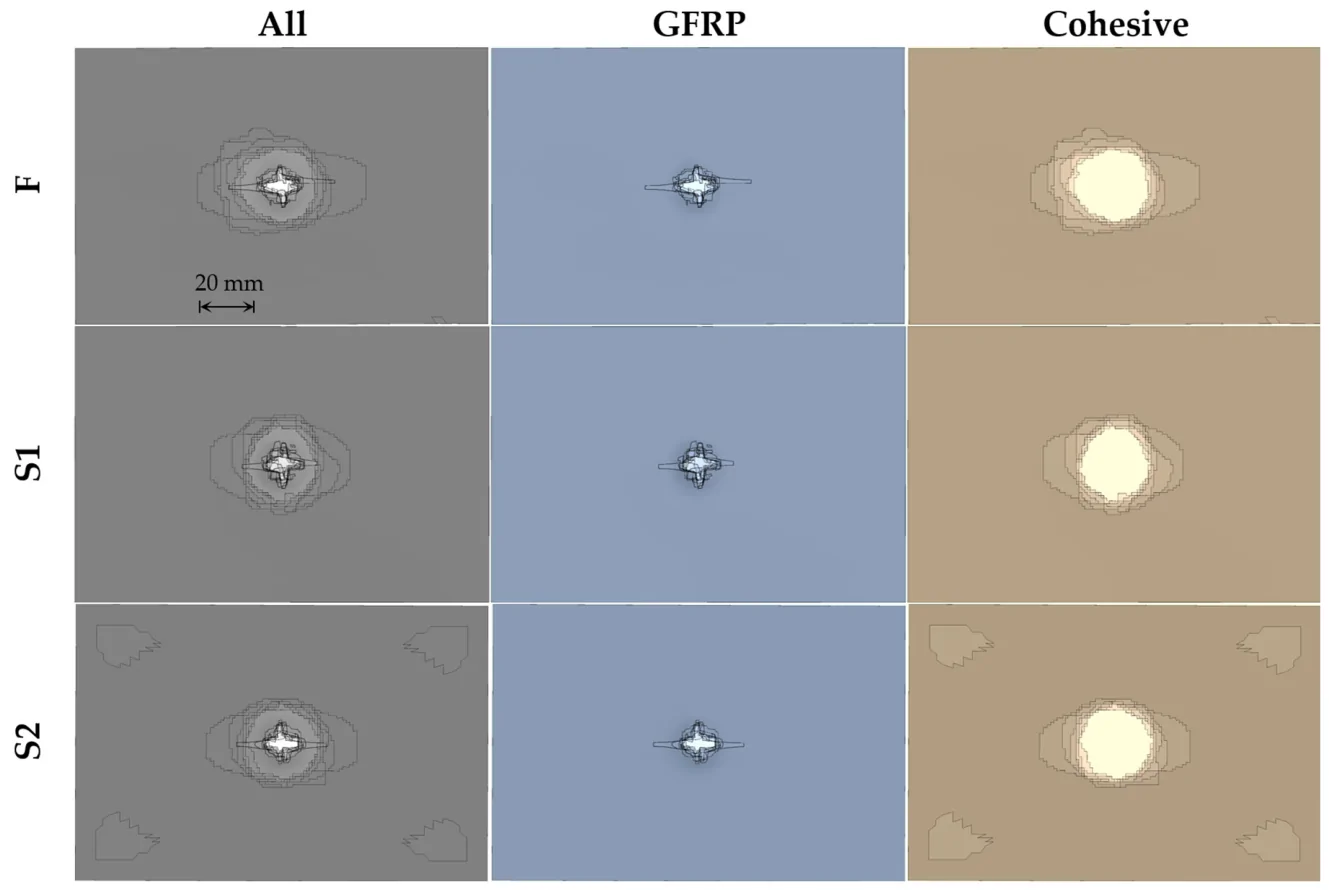
Damage area comparison for FE models under 30 J impact.

To further investigate how boundary condition representation may impact failure prediction, post-impact damage morphology was examined at 30 J impact energy. Figure 13 presents the experimental damage morphology at 30 J for qualitative comparison with the numerical damage predictions shown in Figure 14. From Figure 14, damage distributions are shown for the F, S1, and S2 models, distinguishing between damage in all layers, GFRP layers, and cohesive layers. Differences in the extent and distribution of damage can be observed between the experimental and numerical results. However, the experimental C-scan images and numerical element deletion patterns do not represent identical measures of damage, and the comparison is therefore primarily qualitative.

Although the global impact responses remain largely unchanged between the FE models, examination of the post-impact damage morphology reveals subtle differences in localised failure mechanisms arising from the boundary representation. This indicates that boundary condition representation does not significantly influence the formation of damage.

A distinct difference was observed only in the S2 model, where additional delamination developed near the constrained plate edges despite the absence of direct impact loading. This effect can be attributed to the nodal constraints introduced in the BC representation, which impose artificial stiffness at the periphery of the structure. These constraints generate localised tension in the surrounding elements when global bending occurs during impact. Furthermore, the coarser cohesive elements near the plate boundaries may influence the spatial resolution and localisation of the predicted damage. This combination may contribute to nonphysical delamination near the edges, emphasising that predictive agreement based solely on force or energy histories may overlook important differences in damage morphology. [49]. The observation demonstrates that simplified boundary representations may introduce localised artefacts, emphasising that predictive agreement based solely on force or energy histories may overlook important differences in predicted damage morphology.

To quantify the influence of boundary condition representation, the excess delamination generated outside the impact zone was measured and compared with the total delamination predicted by the F model:

(6) $$\eta_{BC}=\frac{A_{S2}-A_{F}}{A_{F}}\times 100\%$$

where $A_{S2}$ is the area of delamination in S2 model and $A_{F}$ is the area of delamination in the F model. Furthermore, the difference in energy between the S2 and F models was also compared:

(7) $$Energy\;difference=\;\frac{\left|E_{S2}-E_{F}\right|}{E_{F}}\times 100\%$$

where $E_{S2}$ is the absorbed energy in S2 model and $E_{F}$ is the absorbed energy in F model.

**Figure 15:**
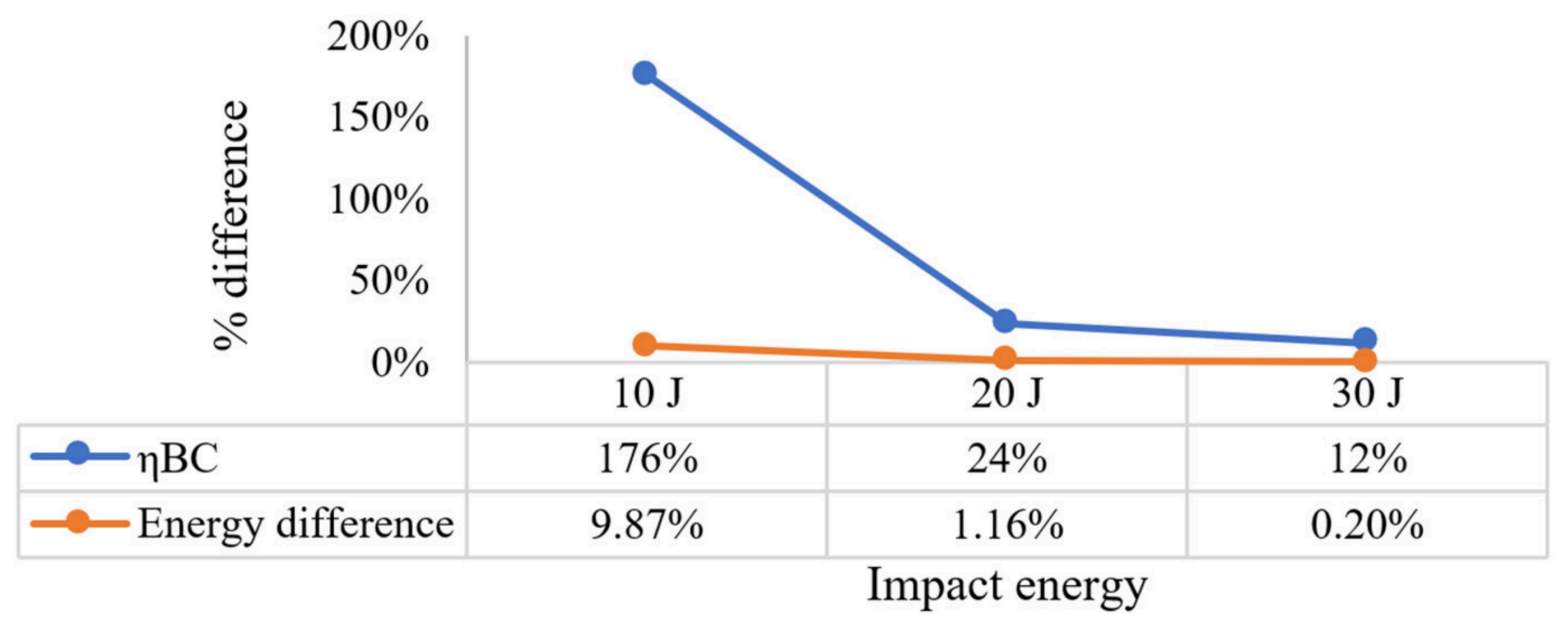
Non-physical delamination of S2 model compared to F.

Figure 15 shown below demonstrates that the influence of BC representation abstraction is inversely related to impact severity. At low impact energies, where impact-induced delamination is relatively small, the stress concentrations introduced by the equivalent nodal constraints contribute a substantial proportion of the predicted damage. As the impact energy increases, impact-induced delamination becomes the dominant failure mechanism, progressively reducing the relative influence of boundary-induced artefacts. These results quantitatively demonstrate that the influence of boundary representation is primarily confined to localised damage prediction, whereas the global structural response remains governed by the impact event itself. The dominant energy dissipation mechanism remains localised beneath the impactor, where the damage evolution is similar between the S2 and F models. The results reveal a separation between global and local response sensitivity. This distinction suggests that the required model detail should be selected according to the response quantity of interest rather than uniformly increasing model complexity for all applications.

### 3.3. Computational Efficiency from BC Representation

This study explores how progressive simplification of boundary conditions affects not only the physical prediction of impact response but also the computational cost associated with FE simulations. The total number of each type of element and the respective simulation times for the F, S1, and S2 models under 10 J, 20 J, and 30 J impact energies are compiled in Table 5.

The S1 and S2 models, which incorporate representative boundary constraints, yield notable reductions in simulation time. S1 and S2 models resulted in average time savings of approximately 20% and 15%, respectively, relative to the F model. The reduction in simulation time of representative boundary conditions is mainly attributed to the removal of contact interaction, rather than the reduction in elements, as rigid body elements do not contribute much towards computational time. This observation confirms that contact interaction, rather than the number of rigid-body elements, dominates the computational cost associated with boundary modelling.

The computational gains from BC simplifications are achieved without significant compromise in predictive response, particularly for global response parameters such as peak force, displacement, and energy absorption. Overall, the results demonstrate that detailed boundary representation is unnecessary when the objective is to predict global impact response. However, realistic support modelling remains important when localised boundary damage or peripheral delamination forms part of the analysis objective [38].

## 4. Results and Discussion—Laminate Representation

### 4.1. Influence of Laminate Representation on Impact Response

**Figure 16:**
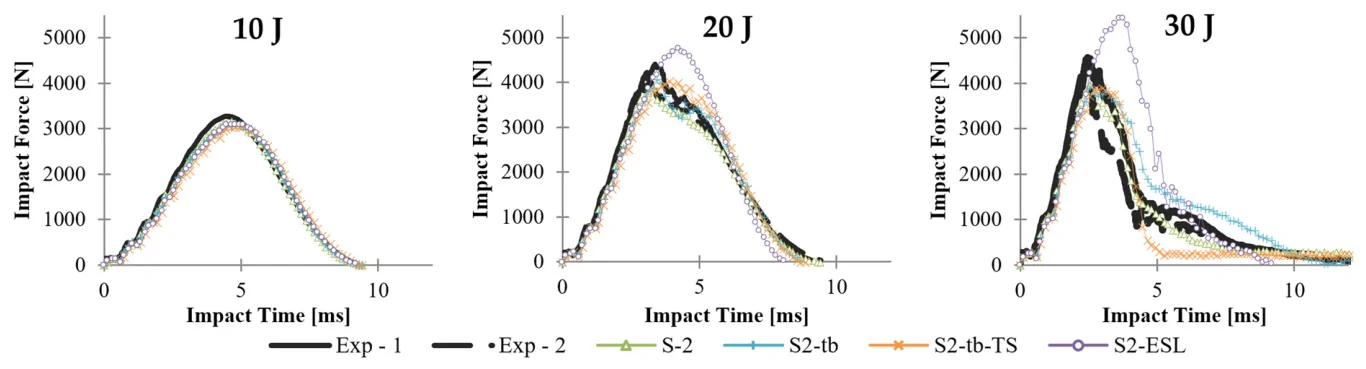
Force-time curve for experiments and FE models with different laminate representations. (colour should be used).

**Figure 17:**
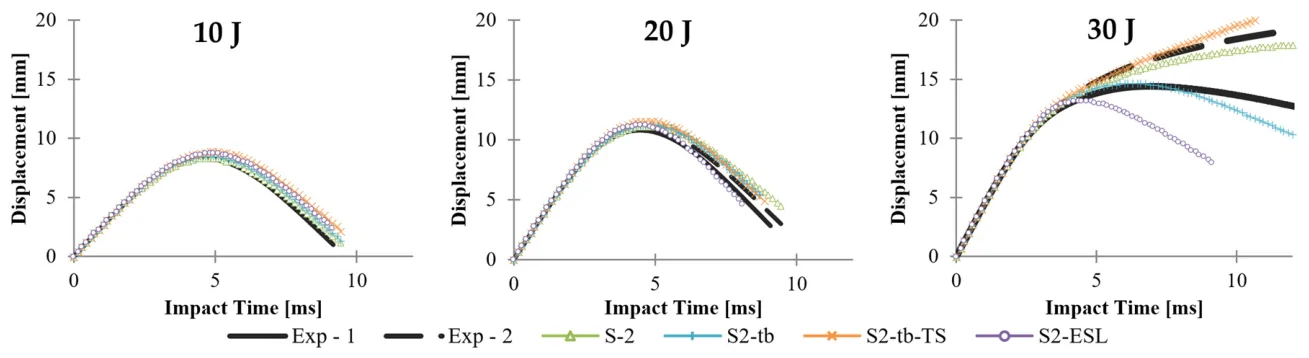
Displacement-time curve for experiments and FE models with different laminate representations. (colour should be used).

**Figure 18:**
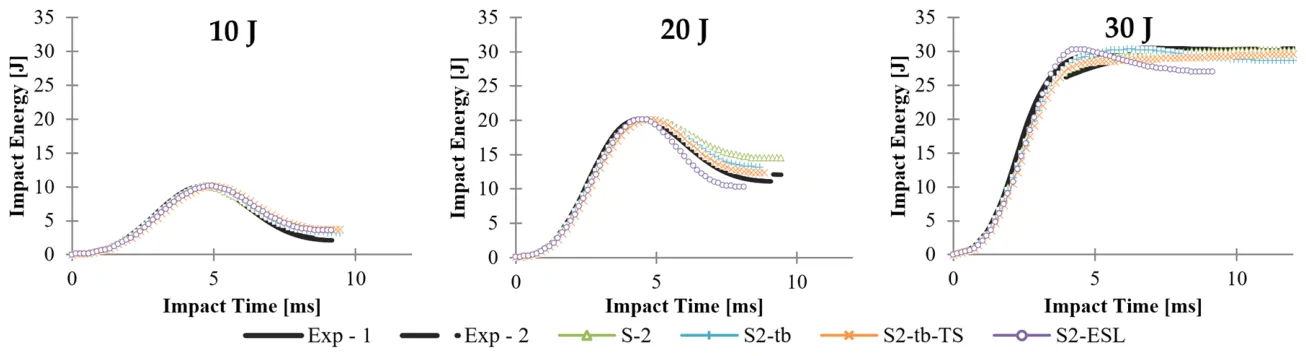
Energy-time curve for experiments and FE models with different laminate representations. (colour should be used).

**Figure 19:**
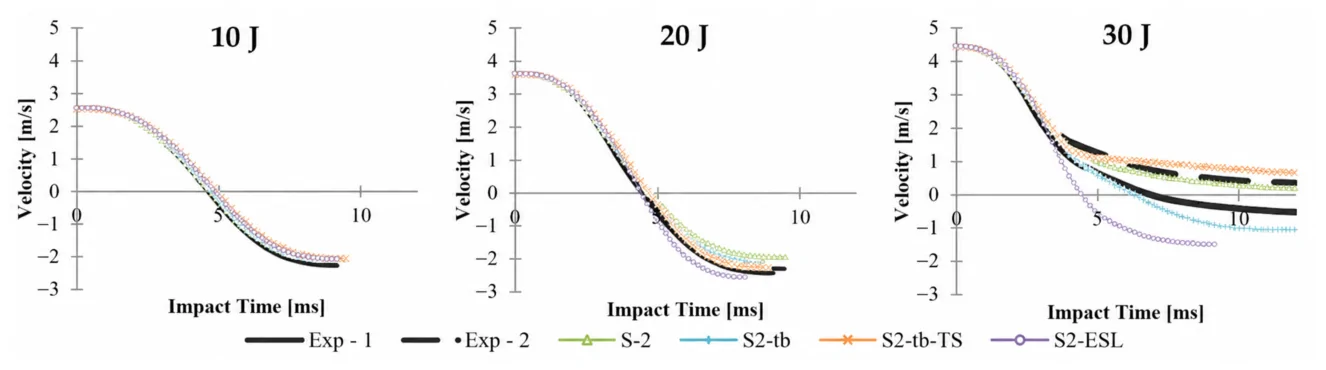
Velocity-time curve for experiments and FE models with different laminate representations. (colour should be used).

Having established the influence of boundary representation, the second stage of the representation examines how laminate representation affects global impact behaviour. Unlike the previous section, the boundary conditions remain unchanged, allowing the isolated influence of interface formulation and through-thickness discretisation on the impact response to be quantified (Figure 16, Figure 17, Figure 18 and Figure 19).

The close agreement at 10–20 J indicates that the global structural response is primarily governed by the overall laminate stiffness before extensive interlaminar damage develops. Consequently, lower-detail laminate representations remain capable of reproducing the principal impact characteristics within this loading regime while offering a reduced computational time [12].

Once extensive interlaminar damage develops at 30 J, the influence of laminate representation becomes substantially more pronounced because the structural response is no longer governed solely by elastic bending but also by the manner in which stiffness progressively degrades during damage evolution. Unlike cohesive elements, tiebreak interfaces release their load-carrying capacity once the failure criterion is satisfied without reproducing the gradual traction–separation degradation associated with crack propagation. Consequently, the laminate retains an unrealistically stiff post-damage response, producing higher residual force and reduced deformation.

Conversely, the S2-tb-TS model shows minimal residual force after peak, with accompanying increases in displacement, energy, and velocity. The inability of the thick-shell formulation to explicitly resolve through-thickness stress gradients suppresses the interlaminar stress transfer phenomenon, resulting in premature damage localisation and excessive penetration once large out-of-plane deformation develops.

On the other hand, the S2-ESL model formulation remains applicable only while the laminate response is dominated by elastic deformation. Once damage initiation governs the structural response, omission of layer-wise failure mechanisms causes a substantial overestimation of structural stiffness and impact resistance. Hence, equivalent single-layer models are very limited in studies involving damage.

**Figure 20:**
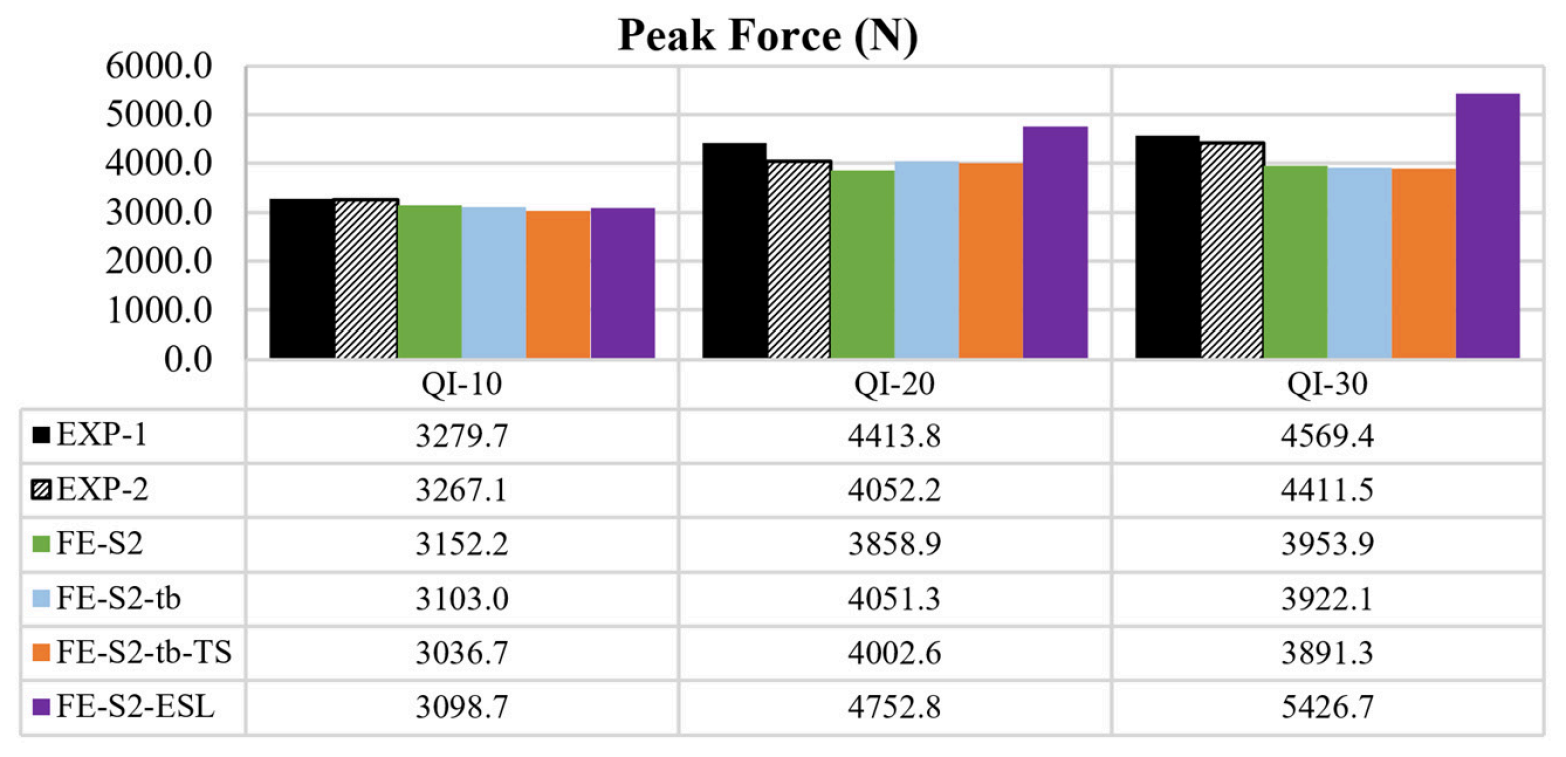
Peak Force for experiments and FE models with different laminate representations.

**Figure 21:**
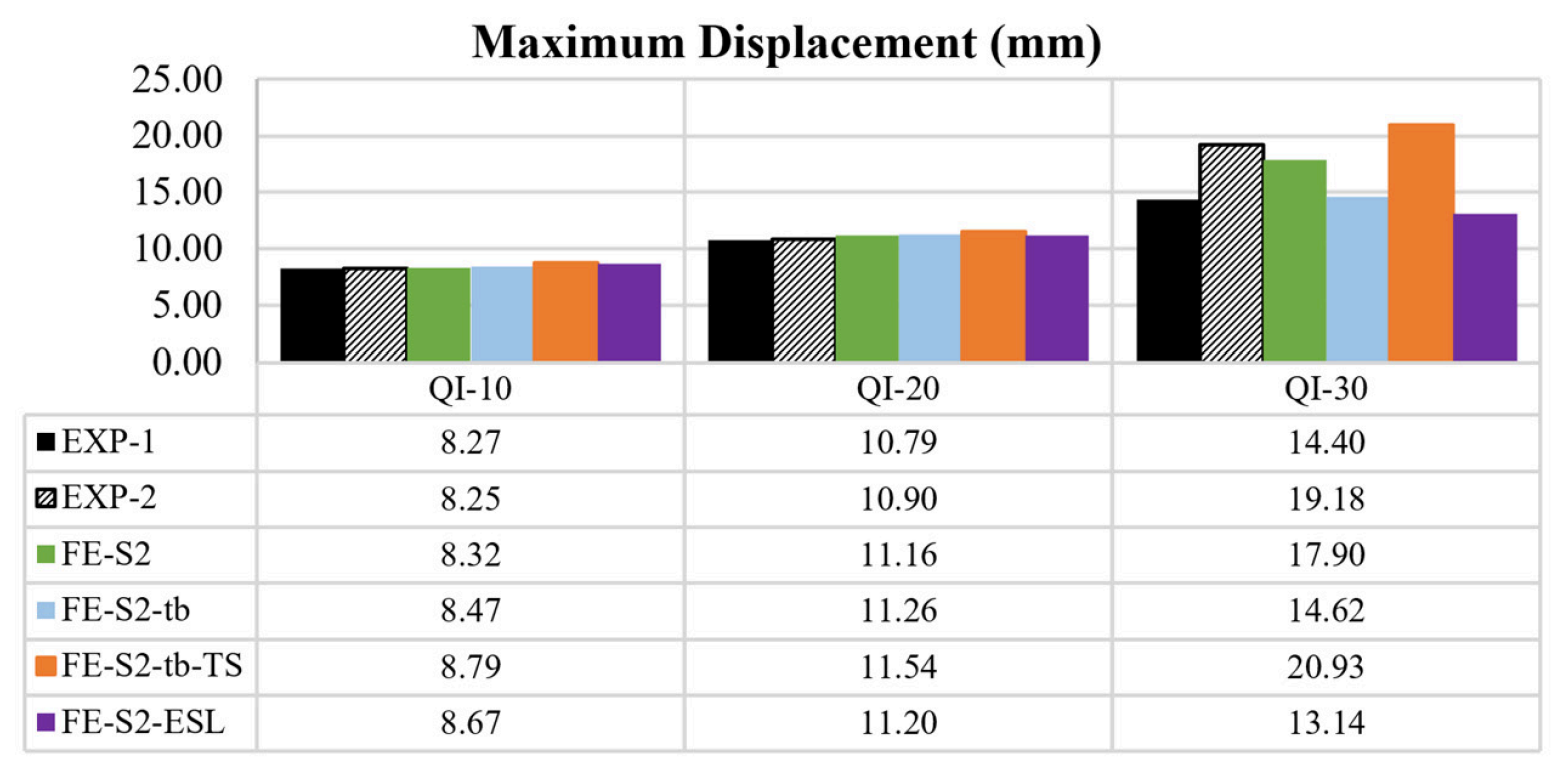
Maximum displacement for experiments and FE models with different laminate representations.

**Figure 22:**
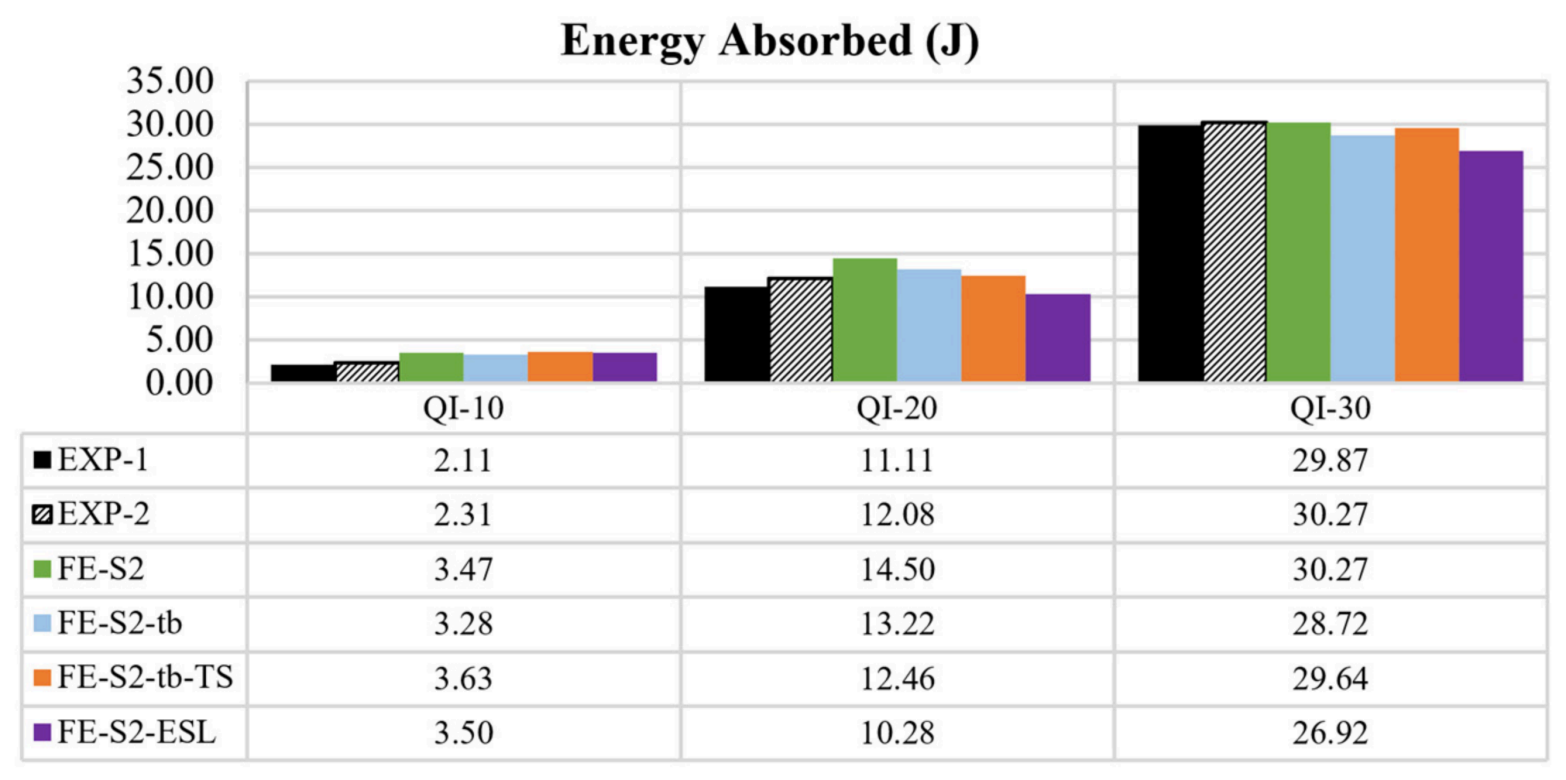
Energy absorbed for experiments and FE models with different laminate representations.

**Figure 23:**
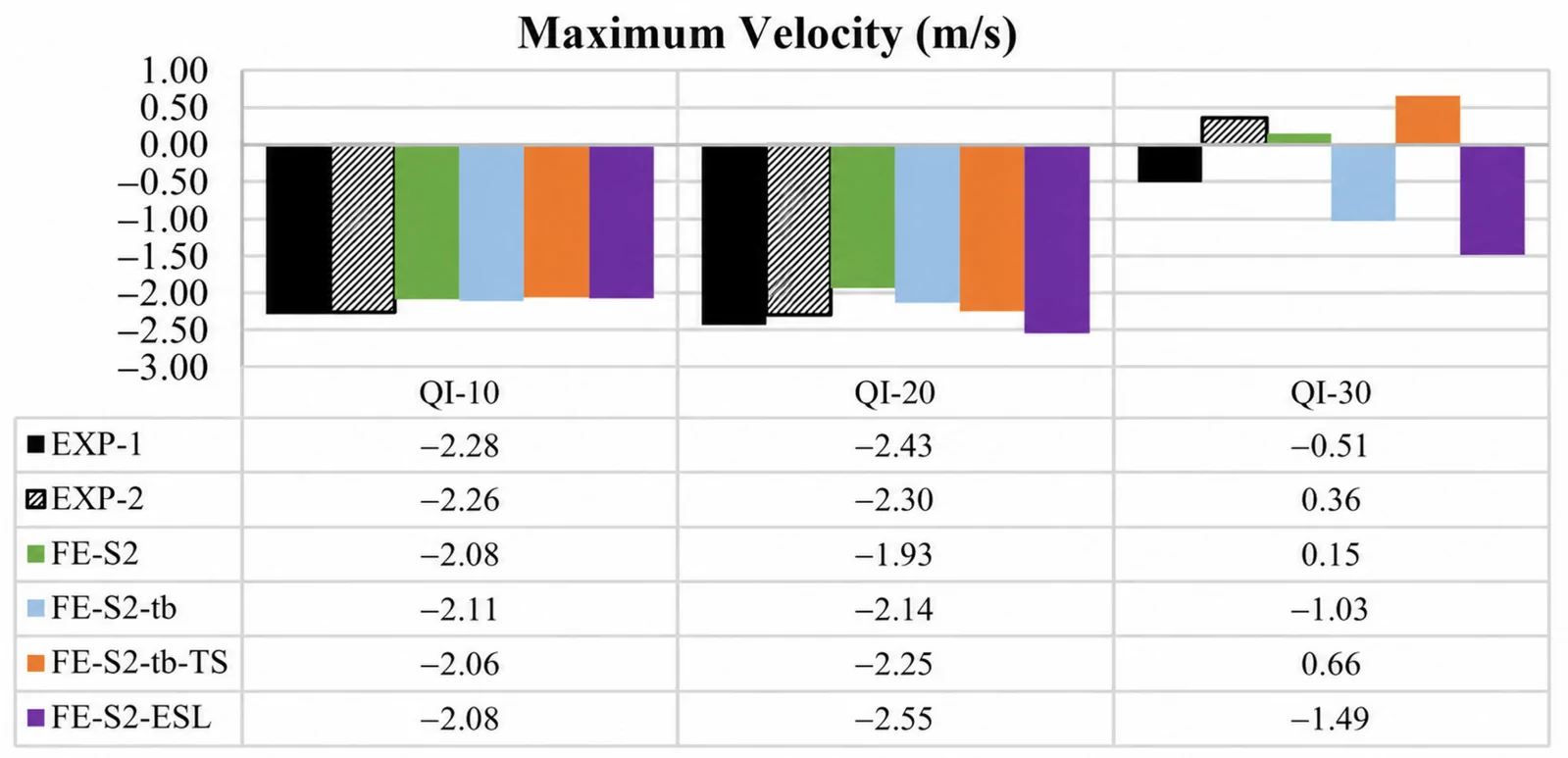
Maximum velocity for experiments and FE models with different laminate representations.

To complement the time history curves, peak values of key impact metrics—peak force, maximum impactor displacement, energy absorbed, and maximum rebound velocity—are extracted and summarised in Figure 20, Figure 21, Figure 22 and Figure 23. The quantitative metrics reinforce the trends observed in the response histories, confirming that laminate abstraction has minimal influence before extensive damage develops but progressively affects all global response quantities once damage evolution becomes the dominant mechanism. S2-ESL has much higher peak force at 20 J and 30 J due to higher plate stiffness, as no damage occurs.

All models predict displacements within the bounds of Exp-1 and Exp-2 at 10 to 20 J (Figure 21). At 30 J, S2 falls well between Exp-1 and Exp-2. S2-tb remains within the experimental range closer to the lower bound, reflecting its restricted post-peak deformation. S2-tb-TS exceeds the experimental upper limit, likely due to the inability of thick shells to capture out-of-plane resistance and stress localisation sufficiently, which was highlighted in the findings by Thorsson et al. [34]. Maximum displacement for equivalent single layer (S2-ESL) shell elements is much lower than acceptable due to its higher resistance, confirming that the plate does not lose any stiffness until damage occurs in all layers.

By comparing the energy absorbed (Figure 22), the models all slightly over-predict energy absorption at 10 and 20 J, albeit within an acceptable range. The only notable exception is the S2-ESL shell model at 20 J, showing lower energy absorption due to lack of damage. Although the absorbed energies of S2, S2-tb, and S2-tb-TS converge at 30 J, this apparent agreement masks fundamentally different energy dissipation mechanisms. Similar total energy absorption therefore does not necessarily imply similar damage evolution.

The maximum velocity highlights the most notable divergence (Figure 23), especially at 30 J. Maximum rebound velocity proved to be the most sensitive global response quantity, reflecting differences in post-damage stiffness recovery between the laminate representations.

Overall, the results reveal that laminate representation exhibits a clear energy-dependent influence on predictive capability. At low to moderate impact energies, where structural response remains stiffness-dominated, reduced-detail laminate (S2-tb and S2-tb-TS) representations adequately reproduce the global impact response. Once extensive damage develops, however, the representation of progressive interlaminar failure becomes essential.

### 4.2. Effects of Interface Representation on Damage Morphology

**Figure 24:**
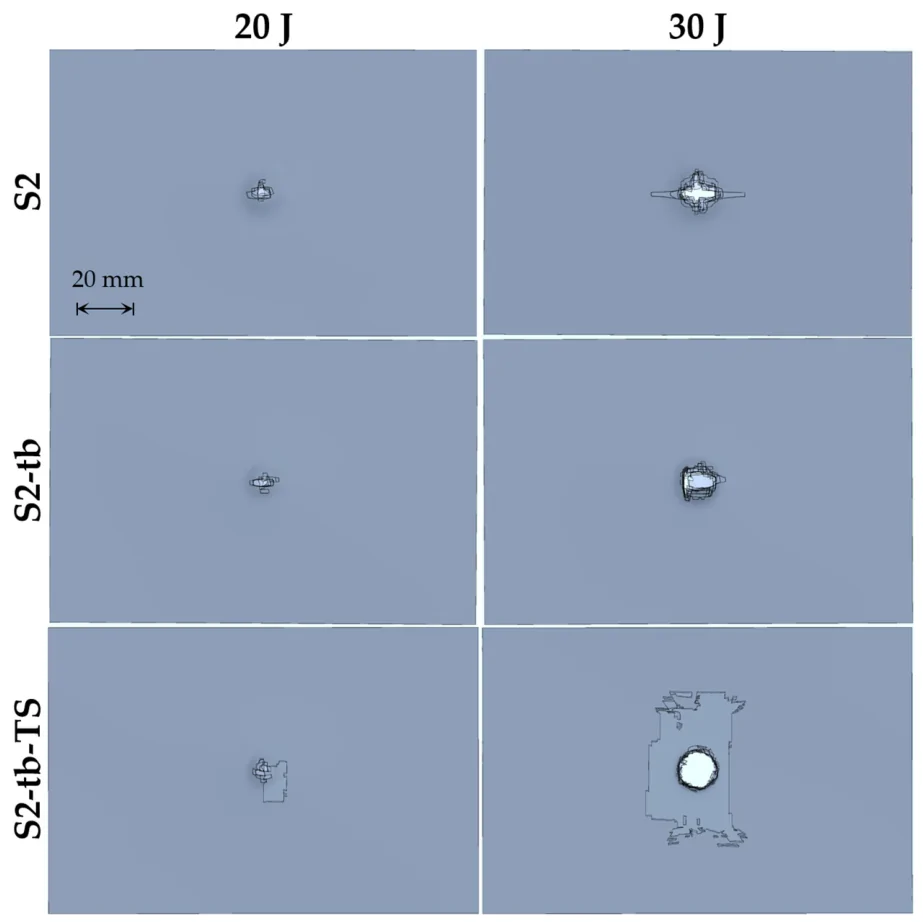
Damage morphology comparison (deleted solid elements of GFRP layers only) for FE models with different laminate representations.

Damage morphologies of the S2-based models were assessed to evaluate their ability to replicate failure patterns under impact (Figure 24). No meaningful visible damage in the form of element deletion was observed in the single-shell model (S2-ESL); therefore, it was not included in this section. This section focuses solely on intralaminar damage in the GFRP layers, as represented by deleted solid elements. Interlaminar delamination is excluded from the displayed damage morphologies, although it is modelled using cohesive elements in the S2 model and tiebreak contact in S2-tb and S2-tb-TS. The analysis is performed only for 20 J and 30 J impact energies, as no discernible damage was observed at 10 J. The experimental damage images are provided in Section 3.2 as reference; however, direct comparison is not considered appropriate, as the numerical images exclude interlaminar delamination.

Differences in laminate representation become more evident when examining the post-impact damage morphology than when comparing global impact responses. Cracks propagating along both longitudinal (0°) and transverse (90°) directions are consistent with fibre orientations and expected matrix cracking. In contrast, S2-tb displays an asymmetric damage mode, with cracks propagating only along the 0° (longitudinal) direction, suggesting that tiebreak contact fails to simulate the full delamination or transverse cracking behaviour typically captured by cohesive elements. Although S2-tb reproduces global force histories, the model concentrates energy dissipation at interface failure initiation rather than allowing progressive crack growth. Meanwhile, S2-tb-TS reveals a non-physical damage localisation, where deletion occurs predominantly in a singular bottom layer, forming a localised yet non-negligible area [34]. This behaviour is attributed to premature element deletion due to out-of-plane stress concentrations, which thick shell elements are not well-equipped to resist.

Under 30 J impact, the deviations become more pronounced. The S2 model produces a distinct cross-shaped failure with a relatively broad damage footprint, reflective of realistic fibre fracture and matrix cracking interactions as well as delamination. In contrast, the S2-tb model exhibits a localised, irregularly shaped hole without the typical crack branching or propagation seen in S2. This aligns with findings by Long et al. [14], where the absence of cohesive layering led to unrealistic failure zones. Without explicit cohesive interfaces and layer-wise stress resolution, progressive crack branching cannot develop naturally, causing failure to localise into an unrealistically compact puncture zone. For the S2-tb-TS model, the damage becomes even more severe. A large circular hole, approximately matching the impactor diameter, fully penetrates through the laminate, suggesting that the entire GFRP layup fails under insufficient out-of-plane resistance. The thick shell model cannot represent through-thickness stresses or interlaminar shear. Furthermore, the bottom-most layer displays an abnormally wide damaged area, indicating that the stress concentration and lack of interlaminar stress modelling led to exaggerated and non-physical element deletion. The damage morphology demonstrates that different laminate representations may produce comparable global impact responses while fundamentally altering the predicted failure mechanisms.

### 4.3. Computational Efficiency from Laminate Representation

The computational efficiency of each abstraction based on S2—namely, S2-tb (with tiebreak contact) and S2-tb-TS (with both tiebreak contact and thick shell elements)—was compared across 10 J, 20 J, and 30 J impact scenarios. Along with the total number of each element type in each model, the simulation times for all cases are summarised in Table 6, providing a direct benchmark against the S2 model.

The S2-tb model demonstrates substantial reductions in computational time. At 10 J, the model represented a 79.6% reduction; at 30 J, 59.3%. Without the cohesive elements, the S2-tb model is much more efficient. This observation is consistent with previous reports [14] identifying cohesive-zone formulations as the principal contributor to computational cost in impact simulations.

The S2-tb-TS model, which further reduces the GFRP laminates with thick shell elements, shows an increase in computational time. Particularly, at 30 J, simulation time sharply increases to 9 h 40 m, even longer than the S2 model. Post-processing of the S2-tb-TS model reveals severe local distortion around the perforation front, including hourglass-type deformation and local negative-volume warnings before element deletion. Although the simulation reached normal termination, these effects resulted in significant timestep reduction and increased computational cost. This result indicates a limitation of thick-shell element formulation for severe through-thickness perforation of high-damage impact conditions.

It is expected that the S2-ESL model would massively reduce computational time, due to a much lower number of elements in the singular layer of shell elements. Combined with the fact that no visible damage occurred for S2-ESL even at high impact energies, the computational time remains at around 30 min.

While these results clearly indicate substantial computational savings, especially for low to moderate impact energies, the gains come at the cost of reduced damage prediction, as previously discussed in Section 4.2. S2-tb, despite being efficient and simulating impact damage at lower energies, fails to replicate the full failure progression at 30 J, where delamination and crack propagation are too concentrated into localised punctures. S2-tb-TS becomes computationally inefficient at high energy due to the inability of thick shell elements to manage complex failure modes, resulting in instability and inflated runtimes. Consequently, reduced-detail laminate representations are best suited for structural analyses where the primary objective is prediction of global response rather than detailed damage evolution. Typical examples include impact-damaged structures subsequently subjected to compression-after-impact, buckling, or combined loading.

## 5. Modelling Strategy Assessment and Engineering Implications

Table 7 summarises the recommended modelling strategies according to predictive capability, computational efficiency, and the intended engineering application. The comparative assessment allows the relative influence of individual modelling assumptions on impact predictions to be evaluated. The results demonstrate that different response quantities require different levels of model detail. Global impact responses, including contact force, displacement, absorbed energy, and impactor velocity, remained largely insensitive to boundary abstraction and simplifications in laminate representation. In contrast, localised damage morphology was considerably more sensitive to interface formulation and through-thickness discretisation, as accurate delamination prediction requires progressive traction transfer and adequate resolution of interlaminar stresses. Consequently, increasing model detail does not uniformly improve all prediction quantities, and the required modelling complexity should be selected according to the engineering objective rather than applied indiscriminately.

From an engineering perspective, boundary abstraction provides a practical reduction in computational cost with minimal influence on global response, while tiebreak interfaces offer substantial additional efficiency when detailed delamination evolution is not the primary objective. Conversely, thick-shell and equivalent single-layer formulations become unsuitable once progressive damage governs the structural response because they cannot adequately reproduce interlaminar failure mechanisms. The proposed strategies therefore provide practical guidance for selecting an appropriate level of model detail for different engineering applications, ranging from preliminary impact assessment to detailed damage analysis and subsequent structural evaluations such as compression-after-impact, buckling, or impact-damaged thin-walled structures. For larger-scale composite structures, S2-ESL may be suitable for global structural response analysis, while S2 offers a balance between computational efficiency and damage prediction. S2-tb may be considered for preliminary impact assessments, whereas F and S1 remain preferable for detailed local damage analysis. However, their applicability to full-scale structures requires further validation. Although the present conclusions are based on one laminate configuration and material system, the proposed modeling guidelines may provide a basis for application to other composite structures and modeling approaches.

## 6. Conclusions

This study systematically evaluated finite element modeling strategies to investigate how boundary conditions and laminate representation influence the predicted impact response, damage evolution, and computational efficiency of low-velocity impact simulations of thin GFRP laminates. The key conclusions are:

1. Alternative boundary representations using nodal constraints with appropriate degrees of freedom and placement were shown to be highly effective across all investigated impact energy levels, resulting in deviations of less than 15% in key impact metrics. This approach also reduced computational cost by approximately 15%. Boundary representation primarily influenced localised peripheral damage, whereas its effect on global impact response remained small. The relative contribution of boundary-induced delamination progressively diminished with increasing impact energy, as impact-driven damage became the dominant failure mechanism.
2. Tiebreak contact definitions provided substantial computational savings, reducing simulation time by up to 70%, and were suitable for preliminary studies at lower and intermediate impact energies. However, they were less capable of accurately reproducing delamination behaviour, especially under high-damage or perforation scenarios. Their application should therefore be limited to analyses where global impact response is of primary interest, and detailed interlaminar damage evolution is not required.
3. Reducing through-thickness discretisation using thick-shell elements proved unsuitable for the investigated thin laminates subjected to severe out-of-plane impact because the formulation could not adequately resolve the interlaminar stress state required for progressive damage evolution. Although the equivalent single-layer formulation provided substantial computational savings, it remained applicable only when progressive damage mechanisms were not the governing response.
4. The required level of model detail should be selected according to the response quantity of interest. Alternative interface representations are appropriate for predicting global impact behaviour, whereas detailed cohesive modeling is preferable whenever the prediction of progressive delamination and perforation is required.
5. The proposed alternative modeling strategies demonstrate that increasing model detail does not uniformly improve all predictions. Instead, different modeling assumptions govern different aspects of structural response, providing a rational basis for selecting an appropriate level of model detail according to the engineering objective. This study provides practical guidelines for similar modeling problems, including channel and cylindrical sections, where fully resolved impact models require considerable computational time. A lower level of model detail is particularly suited to subsequent structural analyses, such as compression-after-impact, buckling, or preloaded structures containing BVID, provided that the relevant damage mechanisms are adequately represented, whereas the detailed reference model remains appropriate for detailed damage assessment.
6. The study demonstrates that agreement in global impact metrics alone is insufficient to establish the adequate modelling strategy. Different numerical representations may produce nearly identical force, displacement, and absorbed energy histories while predicting substantially different internal damage morphologies, emphasising the importance of selecting predictive metrics according to the intended application. Future work may investigate the applicability of these modelling strategies to more complex composite geometries, such as channel and cylindrical sections, with particular emphasis on post-impact structural behaviour under compression and buckling.

## Figures and Tables

**Table 3:** Maximum reported hourglass-to-internal energy ratios (HG/IE) for different models and impact energies.

Model	10 J	20 J	30 J
F	6%	3%	4%
S1	3%	3%	3%
S2	4%	3%	2%
S2-tb	7%	6%	6%
S2-tb-TS	3%	4%	-
S2-ESL	0%	0%	0%

**Table 4:** Levels of modelling strategies investigated in the present study.

Model	Boundary Condition	Delamination Model	Ply Representation
F	Rubber + frame	Cohesive	Solid
S1	4-node constraints + frame	Cohesive	Solid
S2	Distributed nodal constraints	Cohesive	Solid
S2-tb	Same as S2	Tiebreak	Solid
S2-tb-TS	Same as S2	Tiebreak	Thick shell
S2-ESL	Same as S2	None	Equivalent single layer

**Table 1:** Unidirectional GFRP lamina properties used in enhanced composite damage model [39,43].

*ρ* [kg/m^3^]	*E*_1_ [GPa]	*E*_2_ [GPa]	*G*_12_ [GPa]	*G*_23_ [GPa]	*G*_31_ [GPa]	*ν*_12_ [-]
1800	38.5	8.1	2.6	3.1	2.6	0.27
*T*_1_ [MPa]	*C*_1_ [MPa]	*T*_2_ [MPa]	*C*_2_ [MPa]	*S*_12_ [MPa]		
792	679	119	71	96		
*TFAIL*	*ALPH*	*BETA*	*SOFT*	*FBRT*	*YCFAC*	
1 × 10^−9^	0.1	1.0	0.57	0.5	1.2	
*DFAILT*	*DFAILC*	*DFAILM*	*DFAILS*	*EFS*		
0.617	−0.529	0.441	-	-		

**Table 2:** The interlaminar delamination properties in the cohesive zone model [45,46,47].

*ρ* [kg/m^3^]	*G_IC_* [N/m]	*G_IIC_* [N/m]	*σ_max_* [MPa]	*τ_max_* [MPa]	*E_N_* [GPa/mm]	*E_T_* [GPa/mm]
1800	340	2080	20	40	1620	1620

**Table 5:** Simulation time comparison for FE models with different boundary representations.

	GFRP Elements	Cohesive Elements	Rigid Body Elements	10 J	20 J	30 J
F	44,800	39,200	51,680	10 h 25 m	11 h 09 m	11 h 09 m
S1			48,640	8 h 24 m	8 h 44 m	9 h 51 m
S2			48,000	9 h 01 m	9 h 15 m	9 h 16 m

**Table 6:** Simulation time comparison for FE models with different laminate representations.

	GFRP Elements	Cohesive Elements	Rigid Body Elements	10 J	20 J	30 J
S2	44,800	39,200	48,000	9 h 01 m	9 h 15 m	9 h 16 m
S2-tb		-		1 h 50 m	2 h 04 m	3 h 47 m
S2-tb-TS		-		2 h 13 m	2 h 32 m	9 h 40 m
S2-ESL	5,600	-		27 m	35 m	33 m

**Table 7:** Modelling guidance summary based on model performance observed in the present study.

Model	Global Response	Damage Morphology	Delamination	Computational Efficiency	Recommended Application
F	Excellent	Excellent	Excellent	Low	Detailed damage assessment
S1	Excellent	Excellent	Excellent	Moderate	General impact analysis
S2	Excellent	Very good	Good	Moderate	Large structural models
S2-tb	Very good	Moderate	Limited	High	Preliminary impact studies
S2-tb-TS	Moderate	Poor	Poor	Moderate	Not recommended
S2-ESL	Moderate	Poor	None	Very High	Global structural response

## Data Availability

The original contributions presented in this study are included in the article/Supplementary Material. Further inquiries can be directed to the corresponding author.

## Supplementary Materials

The following supporting information can be downloaded at: https://www.mdpi.com/article/10.3390/ma19214458/s1, File manuscript-supplementary.zip: LS-DYNA keyword files and Excel spreadsheets supporting the numerical analyses.

## Nomenclature

FE	Finite element
GFRP	Glass fibre reinforced polymer
CFRP	Carbon fibre reinforced polymer
FRP	Fibre reinforced polymer
LVI	Low-velocity impact
BVID	Barely visible impact damage
ASTM	American Society for Testing and Materials
BC	Boundary condition
F	Detailed reference representation
S1	Level 1 boundary representation
S2	Level 2 boundary representation
S2-tb	Level 2 boundary representation with tiebreak contact
S2-tb-TS	Level 2 boundary representation with tiebreak contact and thick shell elements
S2-ESL	Level 2 boundary representation with equivalent single layer shell elements
*a*	width of the specimen
*b*	length of the specimen
*c*	distance between the specimen edge and the rectangular cutout
*D*	diameter of impactor
*t*	thickness of specimen
ELFORM	element formulation
MAT_054	enhanced composite damage model
MAT_138	cohesive mixed mode model
$e_{f}$	tensile fibre failure
$e_{c}$	compressive fibre failure
$e_{m}$	tensile matrix failure
$e_{d}$	compressive matrix failure
$\sigma_{1}$	normal stress in fibre direction
$\sigma_{2}$	normal stress in transverse direction
$\tau_{12}$	in-plane shear stress
β	shear contribution to fibre tensile failure
*ρ*	density
*E_1_*	longitudinal young’s modulus
*E_2_*	transverse young’s modulus
*G_12_*	shear modulus in xy-plane
*G_23_*	shear modulus in yz-plane
*G_31_*	shear modulus in zx-plane
*ν_12_*	Poisson’s ratio in xy-plane
*T_1_*	longitudinal tensile strength
*C_1_*	longitudinal compressive strength
*T_2_*	transverse tensile strength
*C_2_*	transverse compressive strength
*S_12_*	shear strength in xy-plane
$\delta_{I}^{0}$	Mode-I damage initiation separation
$\delta_{N}$	normal direction ultimate displacement
*G_IC_*	Mode-I critical energy release rate
*σ_max_*	normal direction peak traction
*E_N_*	Mode-I penalty stiffness
$\delta_{II}^{0}$	Mode-II damage initiation separation
$\delta_{T}$	tangential direction ultimate displacement
*G_IIC_*	Mode-II critical energy release rate
*τ_max_*	tangential direction peak traction
*E_T_*	Mode-II penalty stiffness
IHQ	hourglass control type
*ALPH, BETA, SOFT, FBRT, YFAC*	MAT054 ply softening parameters
*DFAILT, DFAILC, DFAILM, DFAILS, EFS*	MAT054 failure criteria
*K*	estimated interface stiffness
α	penalty factor for interface stiffness
$E_{3}$	assumed through-thickness Young’s modulus
*HG*	hourglass
*IE*	internal energy
MAT_020	rigid body material model
U_x_	degree-of-freedom in x-direction
U_y_	degree-of-freedom in y-direction
U_z_	degree-of-freedom in z-direction
MATSUM	material energy summary in LS-DYNA
*E_abs_*	absorbed energy
*E_KE,initial_*	initial kinetic energy
*E_KE,final_*	residual kinetic energy
$\eta_{BC}$	relative difference in delamination area due to boundary condition representation (%)
$A_{S2}$	predicted area of delamination in S2 model
$A_{F}$	predicted area of delamination in F model
$E_{S2}$	absorbed energy in S2 model
$E_{F}$	absorbed energy in F model
